# Weak functional group interactions revealed through metal-free active template rotaxane synthesis

**DOI:** 10.1038/s41467-020-14576-7

**Published:** 2020-02-06

**Authors:** Chong Tian, Stephen D. P. Fielden, George F. S. Whitehead, Iñigo J. Vitorica-Yrezabal, David A. Leigh

**Affiliations:** Department of Chemistry, University of Manchester, Manchester, M13 9PL UK

**Keywords:** Interlocked molecules, Self-assembly

## Abstract

Modest functional group interactions can play important roles in molecular recognition, catalysis and self-assembly. However, weakly associated binding motifs are often difficult to characterize. Here, we report on the metal-free active template synthesis of [2]rotaxanes in one step, up to 95% yield and >100:1 rotaxane:axle selectivity, from primary amines, crown ethers and a range of C=O, C=S, S(=O)_2_ and P=O electrophiles. In addition to being a simple and effective route to a broad range of rotaxanes, the strategy enables 1:1 interactions of crown ethers with various functional groups to be characterized in solution and the solid state, several of which are too weak — or are disfavored compared to other binding modes — to be observed in typical host–guest complexes. The approach may be broadly applicable to the kinetic stabilization and characterization of other weak functional group interactions.

## Abstract (web summary)

Weak interactions between functional groups are often difficult to characterize in host–guest complexes. Here, the authors report the metal-free active template synthesis of a series of rotaxanes that permit a range of kinetically stabilized, weak crown ether-axle interactions to be characterized in solution and the solid state.

## Introduction

The bulky axle end-groups of rotaxanes mechanically lock rings onto threads, preventing the dissociation of the components even if the interactions between them are not strong and attractive^1–3^. In principle the enforced high local concentration of convergent functional groups brought about by such mechanical bonding can stabilize weak non-covalent interactions^4^. In practice such outcomes are rarely observed^4–8^ because most rotaxane syntheses rely upon strong attractive interactions between the building blocks^2,3,9–13^ to promote the rotaxane assembly process. Strong binding modes generally ‘live on’ in the interlocked product, an outcome useful for the design of artificial molecular machinery^2,3,14–16^, whether intended to operate in solution^17^ or when organized on surfaces^18,19^ or within metal-organic frameworks^20,21^, but one that tends to override alternative weaker binding modes that could occur between the components. It is sometimes possible to remove strong template interactions by post-assembly modification, for example by deprotonation of an ammonium unit^22,23^, but this is often not straightforward and can require forcing conditions^23^.

**Fig. 1:**
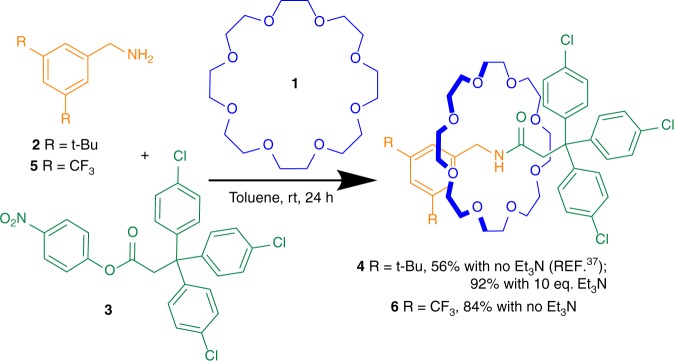
*N*-Acylation active template rotaxane synthesis. The yield of rotaxane is increased by addition of Et_3_N to neutralize the acidic phenolic byproduct of the reaction or the use of less nucleophilic primary amine **5**.

Active template synthesis^24–35^, in which a macrocycle accelerates a strand-forming reaction through the ring cavity, does not require strong pre-association of the starting materials. Although most active template syntheses have been developed from transition metal catalyzed reactions^24–35^, a metal-free active template system was recently discovered^36,37^ in which the addition of primary amines to electrophiles can be significantly accelerated through crown ethers^37^ and related macrocycles^36^ by stabilization of the reaction transition state^38–43^. The reaction of a primary amine and an electrophile in the presence of a crown ether was found^37^ to form [2]rotaxanes by metal-free active template *N*-alkylation, aza-Michael addition or *N*-acylation. In these reactions the crown ether stabilizes developing partial charges in the transition state causing initial rate accelerations of up to 26× through the macrocycle compared with the reaction exo- to the cavity that forms the non-interlocked axle. The *N*-acylation reaction is particularly effective: simply mixing together 1.0 equivalents of each of 24-crown-8 **1**, amine **2**, and activated ester **3** in toluene at room temperature spontaneously assembles amide-axle [2]rotaxane **4** in 56% yield, without the need for any other reagents or excess building blocks (Fig. 1). This potentially offers access to kinetically locked systems with unusual combinations of functional groups on the different components forced into close proximity and a 1:1 stoichiometry. The interaction of the groups on different components might further be enhanced by the tendency of interlocked architectures to have poorly solvated inner surfaces.

To explore the scope of this unexpected method of rotaxane synthesis, here we carry out a study of the reaction with a series of related electrophiles. After developing an optimized set of reaction conditions, rotaxanes were accessed by crown ether-stabilized formation of (thio)urea, carbamate, sulfonamide, and phosphoramidate/phosphinamide-containing axles. The stabilization of S_N_Ar reactions between primary amines and electron-deficient aryl halides led to rotaxanes with aniline threads. Single-crystal X-ray diffraction of the rotaxanes enabled weak interactions between the crown ether and the newly formed functional groups in the axles to be studied.

## Results

### Optimization of metal-free active template rotaxane synthesis by *N*-acylation

Complete consumption of crown ether **1** does not occur with the experimental protocol originally used for the active template *N*-acylation rotaxane-forming reaction (Fig. 1), even with a fivefold excess of amine **2**. Proton nuclear magnetic resonance (^1^H NMR) showed that rotaxane **4** is initially formed rapidly, but over time its rate of formation slows relative to the background reaction of amine and ester, resulting in increasing amounts of non-interlocked axle. The color change that occurs during the early stages of that reaction suggested that liberation of the yellow 4-nitrophenolate anion^44^ might be inhibiting the formation of rotaxane **4**. We reasoned that 4-nitrophenol, formally the other product of the *N*-acylation reaction, would be deprotonated by **2** and the resulting primary ammonium cation (**2**H^+^) would bind strongly to the crown ether preventing it from participating in the active template reaction. Accordingly, we investigated whether the yield of **4** could be improved by the addition of tertiary amines, which when protonated bind more weakly to crown ethers than primary ammonium salts^45^ (Supplementary Table 1). Pleasingly, addition of 10 equivalents (equiv.) of triethylamine (Et_3_N) led to the formation of rotaxane **4** in 68% yield after 1 h and 92% yield after 24 h. Under these conditions the ratio of rotaxane **4** to non-interlocked axle improved from 8:1 to 17:1 after 24 h, indicating that Et_3_N does not promote aminolysis of the building blocks in the absence of the crown ether. In contrast, the use of a stronger base, 1,8-diazabicyclo[5.4.0]undec-7-ene (DBU), significantly reduced the formation of **4** (10% yield after 1 h) while increasing the amount of non-interlocked axle formed, suggesting that DBU accelerates the reaction of **2** and **3** at the expense of the active template reaction^46–48^.

We next investigated the efficacy of rotaxane formation with less nucleophilic benzylic amines (Supplementary Table 2). Commercially available amine **5**, bearing two CF_3_ substituents, proved the most effective amine tested, with [2]rotaxane **6** formed in 84% yield after 24 h without the need for Et_3_N (Fig. 1), with a rotaxane:non-interlocked axle ratio >100:1 (determined by ^1^H NMR). This remarkable selectivity for acylation through the cavity appears to be a consequence of the background acylation reaction (to form the non-interlocked axle) having an activation energy in the ‘sweet spot’ for active template synthesis: too high for acylation to occur quickly with the less nucleophilic amine (**5**) but low enough that a few kcal mol^−1^ stabilization of the transition state by the crown ether brings about a very significant rate enhancement.

It also proved possible to use more reactive electrophiles with amine **5** (Supplementary Table 3). Rotaxane **6** was obtained in 54% yield from the corresponding acid chloride and in 40% yield when using the 1-hydroxybenzotriazole ester as the electrophile.

### C=O/C=S/SO_2_/P=O electrophile scope

With improved conditions for active template ester aminolysis in hand we investigated whether the type of rotaxanes accessible could be expanded upon using electrophiles based on different, but structurally related, chemical functionality (**7**–**14**, Table 1). The aminolysis of carbamates^49^ follows a similar mechanistic pathway to ester aminolysis: nucleophilic attack at the carbonyl forms a tetrahedral intermediate followed by loss of the leaving group to form urea^50–52^. Accordingly we tested whether carbamate **7** was a suitable electrophile for the metal-free active template reaction. Reaction of **7**, amine **5** and 24-crown-8 **1** in a 1:1:1 ratio, under the standard reaction conditions (without Et_3_N), afforded urea [2]rotaxane **15** in 73% yield (Table 1, entry 1). Urea rotaxane formation was also possible without generating a leaving group byproduct through the use of isocyanate **8**, which gave rotaxane **16** in 55% yield (Table 1, entry 2). The reaction between **5** and **8** proceeded extremely quickly; full conversion of **5** was achieved within 1 min. The corresponding thiourea rotaxane **17** was prepared in an analogous manner from isothiocyanate **9** in 54% yield (Table 1, entry 3).

**Table 1:** Synthesis of [2]rotaxanes from 24-crown-8, amine **5** and C=O/C=S/SO_2_/P=O electrophiles **7**–**14**^a^

^a^Reaction conditions: 1 equiv. each of crown ether **1**, amine **5** and electrophile **7**–**14**, toluene [0.14 M], rt.

^b^Yield of isolated product.

^c^1.5 equiv. of electrophile.

^d^2 equiv. of Et_3_N added.

Carbamate rotaxanes were accessible using common commercially available electrophiles. Activated carbonate **10**, used to form carbamates that can be readily decomposed with fluoride^53^, gave **18** in 83% yield (Table 1, entry 4), while chloroformate **11** (Fmoc-Cl) generated **19** in 70% yield with Et_3_N added to neutralize the HCl product (Table 1, entry 5)^54^. The ability to release the macrocycle from these types of rotaxanes in response to a specific chemical stimulus (stoichiometric fluoride for **18**; catalytic base for **19**) may prove useful for future applications.

Electrophiles containing a heteroatom at the site of nucleophilic attack also proved effective for rotaxane formation. Sulfonyl chloride **12**, a bulky analog of tosyl chloride, reacted with **5** and **1** to give sulfonamide rotaxane **20** in 95% yield (Table 1, entry 6). Diphenyl phosphoryl chloride **13** produced phosphoramidate rotaxane **21** in 90% yield (Table 1, entry 7), while the more reactive diphenyl phosphinic chloride **14** resulted in the formation of phosphinamide rotaxane **22** in a more modest 29% yield (Table 1, entry 8). As the sulfur and phosphorus electrophiles feature chloride leaving groups, in each case Et_3_N was added to neutralize the HCl formally released by the active template reaction.

### Metal-free active template synthesis by *N*-arylation

**Fig. 2:**
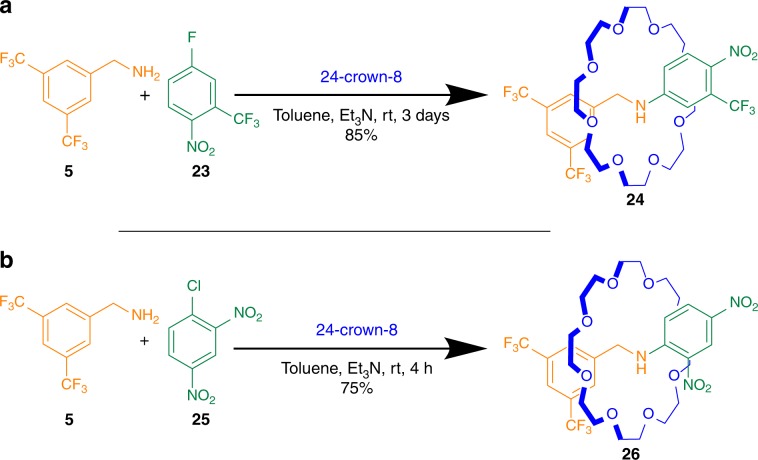
*N*-Arylation active template rotaxane synthesis. Rotaxanes containing aniline axles could be obtained by the S_N_Ar reaction between primary amine **5** and **a** aryl fluoride **23** or **b** aryl chloride **25**.

To further expand on the general applicability of active template rotaxane synthesis with crown ethers, we explored other potential reaction modes. Prompted by a recent report^55^ of crown ether catalysis of S_N_Ar reactions between aryl halides and primary amines, we investigated rotaxane formation by *N*-arylation. This proved effective using different electrophiles: combining amine **5** and 24-crown-8 **1** with aryl fluoride **23** in the presence of Et_3_N produced aniline rotaxane **24** in 85% yield (Fig. 2a), while combining **5** and **1** with aryl chloride **25** formed aniline rotaxane **26** in 75% yield (Fig. 2b). Both rotaxanes were isolated as neutral amines rather than as the corresponding ammonium salts. As the p*K*_a_ values of protonated anilines are readily modulated by changing the aromatic substitution^56^, rotaxanes such as **24** and **26** have the potential to be used as tunable pH-sensitive molecular switches of basicity lower than that of commonly used dibenzylammonium-crown ether systems^3^.

### Crown ether-functional group interactions

Complexes between crown ethers and neutral molecules were first reported by Pedersen nearly 50 years ago^57^, with the majority of examples described to date involving relatively small macrocycles such as 18-crown-6 (refs. ^58–61^). Neutral molecules cannot be fully encapsulated within such small cavities and so the complexes tend to be of a ‘perch’ type. With such binding modes the crown ethers often bind to more than one guest to maximize favorable host–guest interactions and to balance the dipole moments of polar guests. In the solid state discrete 1:1 crown ether–neutral molecule complexes are rare and a range of different binding modes and ratios can sometimes be observed with only minor variations in structure arising from different crystallization conditions^59–61^.

In contrast to such host–guest complexes, the interlocked components of rotaxanes have a strictly defined stoichiometry (usually 1:1), are held in close proximity, and possess limited co-conformational^62^ degrees of freedom^63–71^. In the absence of strong binding between the components weak interactions that are seldom observable in supramolecular complexes can form and significantly influence co-conformation^4,5^. Rotaxanes **4**, **6**, **15**, **17**, and **19**–**22** provide architectures in which functional groups in the axle are mechanically locked through the crown ether ring in a 1:1 stoichiometry, enabling normally weak interactions to be characterized experimentally. Slow evaporation of CH_2_Cl_2_/hexane solutions of **4**, **17**, **21**, and **22**, and of diethyl ether/hexane solutions of **6**, **15**, **19**, and **20** afforded single crystals suitable for X-ray diffraction of rotaxanes containing each of the axle functionalities formed through metal-free active template synthesis.

### X-ray crystal structures of [2]rotaxanes 4 and 6

**Fig. 3:**
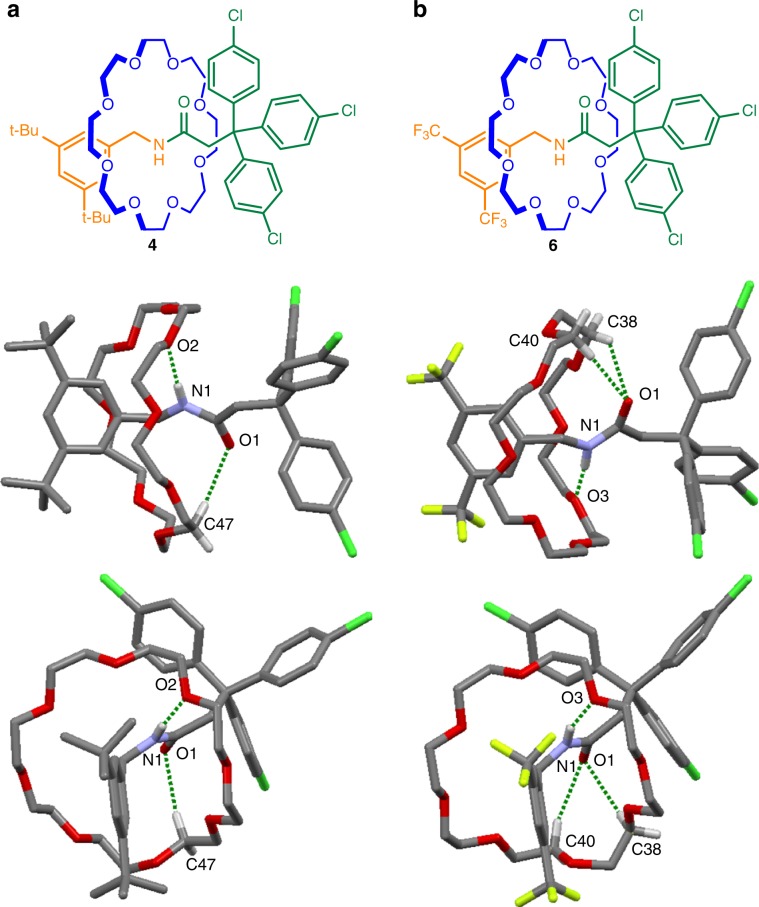
X-ray crystal structures of crown ether–amide-axle rotaxanes. **a** Rotaxane **4**. Hydrogen bond lengths [Å]: O1—H47C, 2.47; O2—H1N, 2.12. Hydrogen bond angles (deg): O1—H47C, 166.3; O2—H1N, 173.1. **b** Rotaxane **6**. Hydrogen bond lengths [Å]: O1—H38C, 2.48; O1—H40C, 2.55; O3—H1N, 2.20. Hydrogen bond angles (deg): O1—H38C, 129.5; O1—H40C, 161.0; O3—H1N, 163.0. NH···O and CH···O hydrogen bonds shown in dark green. Solvate molecules and other hydrogen atoms omitted for clarity.

In structures in the Cambridge Crystallographic Data Centre (CCDC) database, crown ether–amide host–guest binding typically occurs with a 1:2 stoichiometry, with the crown ether oxygen atoms accepting hydrogen bonds from the amide guest^57–60^. In the amide-axle rotaxanes **4** and **6** (Fig. 3) a 1:1 crown ether:amide interaction stoichiometry occurs and in each structure a crown ether oxygen hydrogen bonds to the amide hydrogen atom while CH^….^O hydrogen bonds stabilize the electron density on the amide oxygen atom^72,73^. The O^….^H–N hydrogen bond in **4** is shorter than in **6** (O^….^H distances: 2.12 Å (**4**); 2.20 Å (**6**)), despite the electron-withdrawing CF_3_ groups in **6**. In **4** the oxygen interacts with a single C–H group (O^….^H distance: 2.47 Å), while in **6** three-centered bifurcated hydrogen bonding occurs between the carbonyl oxygen and two C–H groups (O^….^H distances: 2.48 and 2.55 Å)^74^. Such CH^….^O hydrogen bonding is reminiscent of interactions within peptide chains that stabilize protein secondary structure^75^.

### X-ray crystal structures of [2]rotaxanes 15, 17, and 19

**Fig. 4:**
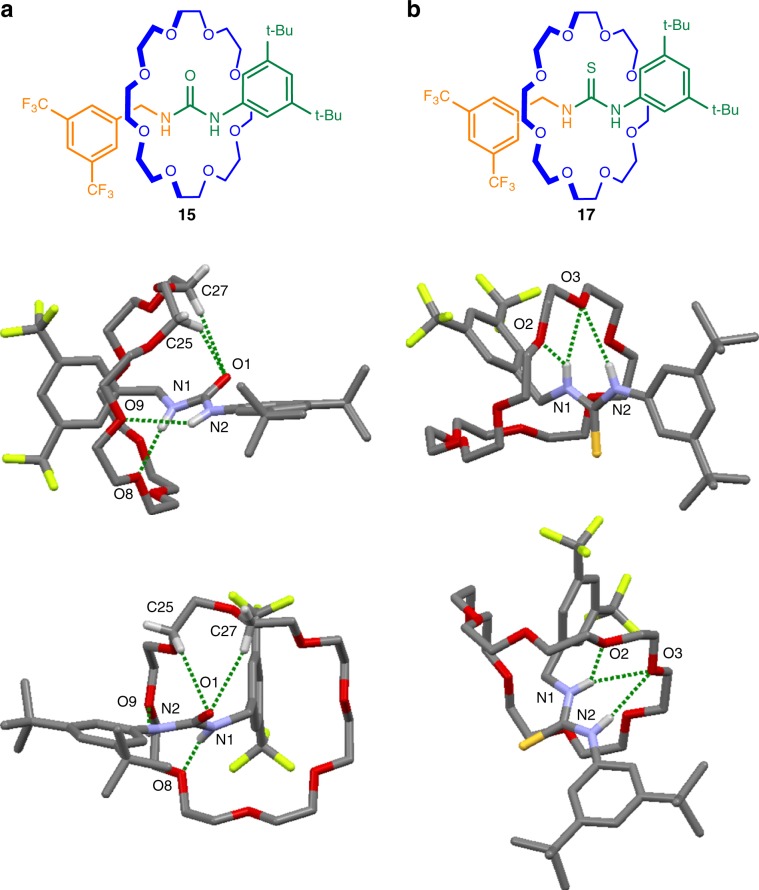
X-ray crystal structures of crown ether urea and thiourea axle rotaxanes. **a** Rotaxane **15**. Hydrogen bond lengths [Å]: O1—H25C, 2.66; O1-H27C, 2.71; O8—H1N, 2.12; O9—H2N, 2.65. Hydrogen bond angles (deg): O1—H25C, 145.4; O1-H27C, 165.0; O8—H1N, 169.7; O9—H2N, 143.2. **b** Rotaxane **17**. Hydrogen bond lengths [Å]: O2—H1N, 2.41; O3—H1N, 2.32; O3—H2N, 2.74. Hydrogen bond angles (deg): O2—H1N, 126.9; O3—H1N, 158.3; O3—H2N, 148.5. NH···O and CH···O hydrogen bonds shown in dark green. Solvate molecules and other hydrogen atoms omitted for clarity.

**Fig. 5:**
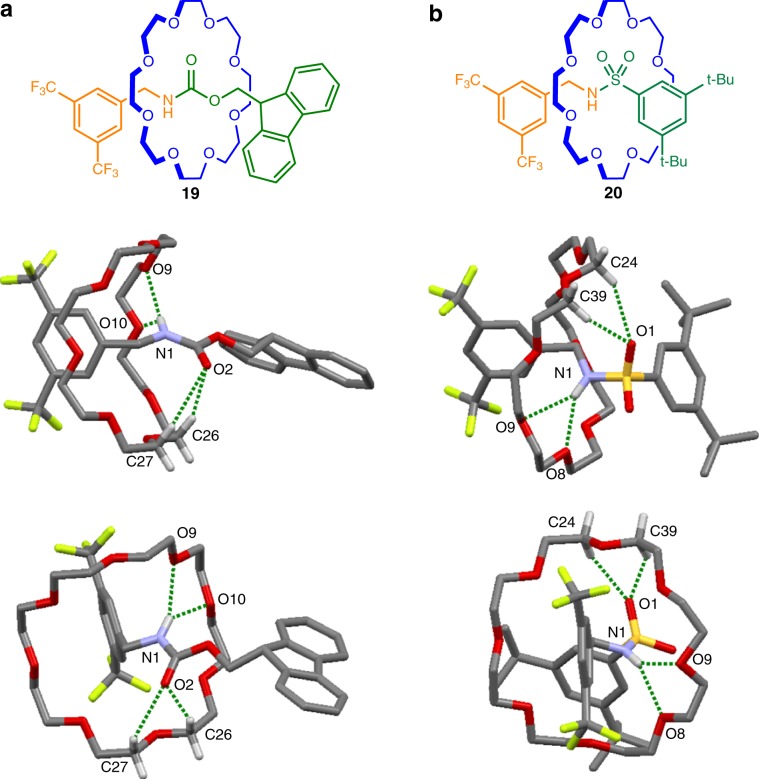
X-ray crystal structures of crown ether carbamate and sulfonamide axle rotaxanes. **a** Rotaxane **19**. Hydrogen bond lengths [Å]: O2—H26C, 2.53; O2—H27C, 2.88; O9—H1N, 2.27; O10—H1N, 2.65. Hydrogen bond angles (deg): O2—H26C, 155.4; O2—H27C, 142.7; O9—H1N, 154.4; O10—H1N, 135.0. **b** Rotaxane **20**. Hydrogen bond lengths [Å]: O1—H24C, 2.50; O1—H39C, 2.64; O8—H1N, 2.31; O9—H1N, 2.61. Hydrogen bond angles (deg): O1—H24C, 149.3; O1—H39C, 144.8; O8—H1N, 145.6; O9—H1N, 144.9. NH···O and CH···O hydrogen bonds shown in dark green. Solvate molecules and other hydrogen atoms omitted for clarity.

Crown ethers and (thio)ureas tend to form complex extended networks in the solid state, with the ratio of host-to-guest varying significantly and unpredictably^57^. In contrast rotaxanes **15**, **17**, and **19** form discrete structures with 1:1 crown ether:urea/thiourea association. Rotaxanes **15** and **17** (Fig. 4) each contain two different types of N–H groups: one nitrogen atom conjugated to an aromatic ring, the other benzylic. In both solid state structures the crown ether forms shorter hydrogen bonds with the benzylic N–H group. In **15** the interaction occurs with a single crown ether oxygen (O^….^H distance: 2.12 Å), while a bifurcated hydrogen bond occurs with two oxygen atoms in **17** (O^….^H distances: 2.32 and 2.41 Å). An additional long hydrogen bond from an ether oxygen to the other NH group is present in both rotaxanes (O^….^H distances: 2.65 and 2.74 Å for **15** and **17**, respectively). A bifurcated hydrogen bond between two crown ether oxygens and the N–H moiety of the carbamate group (O^….^H distances: 2.27 and 2.65 Å) occurs in **19**. The slightly longer O^….^HN bond lengths in **19** (Fig. 5) versus **15** are consistent with the weaker hydrogen bond-donating ability of a carbamate group compared with urea^76,77^. Similar to an amide group, the carbonyl oxygens act as hydrogen bond acceptors from C–H groups of the crown ether in **15** and **19**, with bifurcated hydrogen bonds formed in both cases (O^….^H distances: 2.66 and 2.71 Å for **15**; 2.53 and 2.88 Å for **19**). The sulfur atom in **17** does not engage in a similar interaction reflecting the more modest hydrogen bond basicity of thioureas^78^.

### X-ray crystal structures of [2]rotaxanes 20–22

In the solid state rotaxanes **20**, **21**, and **22** all have rather similar intercomponent interactions to each other. In supramolecular complexes, sulfonamides usually bind to crown ethers through hydrogen bonds donated by the N–H group^79^. In the X-ray crystal structure of **20** the N–H forms a bifurcated hydrogen bond with two crown ether oxygens (O^….^H distances: 2.31 and 2.61 Å; Fig. 5). Another bifurcated hydrogen bond is formed between two C–H groups of the crown ether and a single sulfonamide oxygen (O^….^H distances: 2.50 and 2.64 Å). The crown ether does not adopt a conformation that enables simultaneous interactions with both sulfonamide oxygens^80^.

**Fig. 6:**
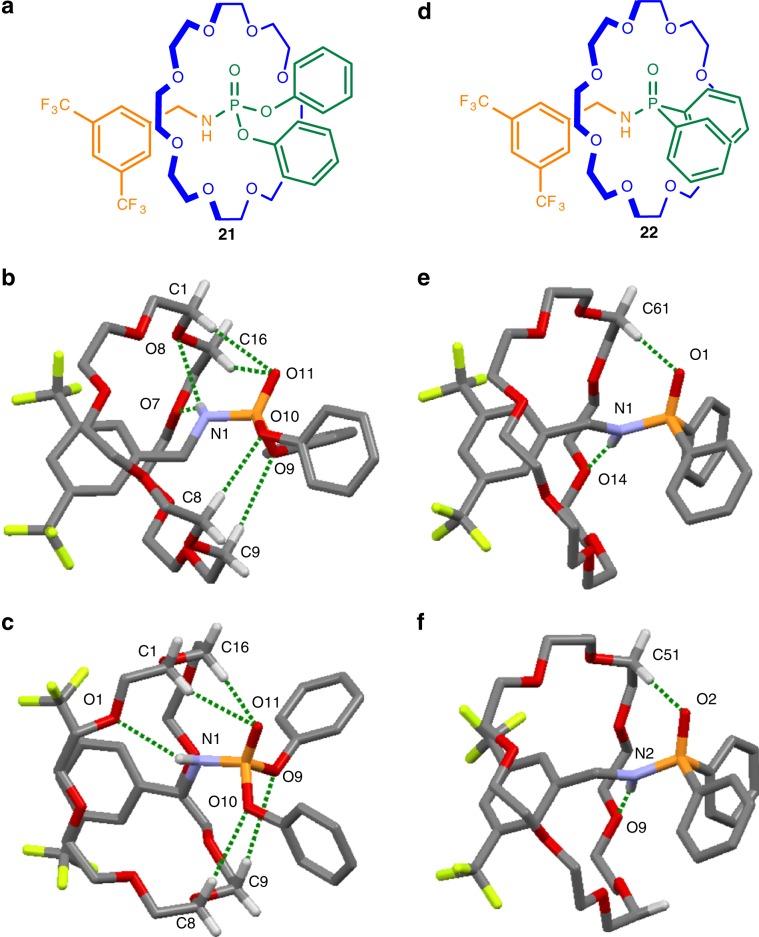
X-ray crystal structures of crown ether phosphoramidate and phosphinate axle rotaxanes. **a** Rotaxane **21**. The hydrogen atom bonded to N_1_ in **21** is disordered over two positions, each structure is shown in (**b**) and (**c**). **b** Hydrogen bond lengths [Å]: O9—H9C, 3.14; O10—H8C, 2.75; O11—H1C, 2.52; O11—H16C, 2.60; O7—H1N, 2.71; O8—H1N, 2.62. Hydrogen bond angles (deg): O9—H9C, 165.3; O10—H8C, 168.2; O11—H1C, 152.5; O11—H16C, 146.1; O7—H1N, 157.7; O8—H1N, 134.0. **c** Hydrogen bond lengths [Å]: O1—H1N, 2.65; O9—H9C, 3.14; O10—H8C, 2.75; O11—H1C, 2.52; O11—H16C, 2.60. Hydrogen bond angles (deg): O1—H1N, 144.2; O9—H9C, 165.3; O10—H8C, 168.2; O11—H1C, 152.5; O11—H16C, 146.1. **d** Rotaxane **22**. Two co-conformations of **22** co-crystallize, each is shown in (**e**) and (**f**). **e** Hydrogen bond lengths [Å]: O1—H61C, 2.39; O14—H1N, 2.36. Hydrogen bond angles (deg): O1—H61C, 171.5; O14—H1N, 174.3. **f** Hydrogen bond lengths [Å]: O2—H51C, 2.37; O9—H2N, 2.41. Hydrogen bond angles (deg): O2—H51C, 172.5; O9—H2N, 175.1. NH···O and CH···O hydrogen bonds shown in dark green. Solvate molecules and other hydrogen atoms omitted for clarity.

Phosphorous analogs of amides often form intermolecular P–O^….^H–N hydrogen bonds in the solid state, driven by the particularly strong hydrogen bond accepting ability of P=O^76,81^. However, as with the other rotaxanes described in this series, intermolecular interactions between axles is inhibited in **21** and **22** by encapsulation of the phosphoramidate and phosphinate units by the crown ether^82,83^. The N–H hydrogen atom in **21** (Fig. 6) is disordered over two positions, while the sp^2^-hybridized oxygen forms a bifurcated hydrogen bond with two crown ether C–H groups (O^….^H distances: 2.52 and 2.60 Å). The sp^3^-hybridized oxygen atoms bound to phosphorous each form relatively long hydrogen bonds with a single C–H group of the crown ether (O^….^H distances: 2.75 and 3.14 Å). Two conformations of **22** co-crystallize, both with a single ether oxygen forming a hydrogen bond with the N–H group (average O^….^H distance 2.39 Å) and a single hydrogen bond between the phosphinyl oxygen atom and a C–H group of the crown ether (average O^….^H distance 2.38 Å).

## Discussion

Metal-free active template synthesis is a simple and versatile method through which to access crown ether rotaxanes with a discrete but diverse set of functionalities in the axle. The rotaxane assembly procedure is exceptionally simple, requiring only mixing of a crown ether, amine, and electrophile in toluene. All of the building blocks used in this paper are either currently commercially available or, in the case of amine **2**, ester **3**, carbamate **7**, isothiocyanate **9**, and sulfonyl chloride **12**, accessible in a single synthetic step. The rotaxane-forming reactions can be performed using a 1:1:1 stoichiometry of the three building blocks, in some cases generating rotaxanes in yields as high as 95% with >100:1 rotaxane:axle selectivity. In addition to being a simple and effective route to a wide range of rotaxanes the strategy enables 1:1 interactions of crown ethers with various functional group types to be characterized, several of which are too weak—or are disfavored compared with other binding modes—to be observed in conventional host–guest complexes. As of 23 September 2019 the CCDC contained >2000 X-ray crystal structures featuring crown ethers. The structures reported in this paper include the first few examples of crown ether–alkylsulfonamide, crown ether–phosphoramidate, crown ether–phosphonate, and crown ether–carbamate interactions, and only the second examples of crown ether–primary amide, crown ether-substituted urea, and crown ether-substituted thiourea interactions. Active template synthesis, essentially the use of catalysis to form and kinetically trap an unstable assembly, should prove to be a generally applicable tool for revealing weak functional group interactions. The difficulties in characterizing weak interactions was recently highlighted by Colizzi et al.^84^: “…intramolecular hydrogen bonds are widespread in biological molecules and are crucial in the design of new drugs and materials, including supramolecular machines… Unfortunately, the characterization of intramolecular H-bonds (and their utilization) is still limited, most likely as a consequence of the complexities that hamper the interpretation of experimental data obtained for large and flexible entities.” The ability to kinetically stabilize modest strength hydrogen bonding modes is another noteworthy consequence of the mechanical bond^3^.

## Methods

### Synthesis of rotaxanes

The synthesis and characterization of the rotaxanes and building blocks are provided in the Supplementary Methods. Representative synthesis of rotaxane **15**: To a stirring solution of **1** (49 mg, 0.14 mmol, 1.0 equiv.) and **5** (34 mg, 0.14 mmol, 1.0 equiv.) in toluene (1.0 mL) was added **7** (52 mg, 0.14 mmol, 1.0 equiv.). The mixture was stirred for 1 h and then concentrated under reduced pressure. Flash chromatography of the residue (SiO_2_, EtOAc/petroleum ether 1:5 then CH_2_Cl_2_/MeOH 50:1) afforded rotaxane **15** as a colorless solid (85 mg, 0.10 mmol, 73%).

## Supplementary information

Supplementary Information

## Data Availability

The authors declare that the main data supporting the findings of this study are available within the article and its Supplementary Information files. Extra data are available from the corresponding author upon request. The X-ray crystallographic coordinates for structures reported in this study have been deposited at the CCDC, under deposition numbers CCDCs 1907190-1907197. This data can be obtained free of charge from The Cambridge Crystallographic Data Centre via www.ccdc.cam.ac.uk/data_request/cif).

## References

[1] Schill, G. *Catenanes, Rotaxanes and Knots* (Academic Press, New York, 1971).

[2] Sauvage, J.-P. & Dietrich-Buchecker, C. O. (eds). *Molecular Catenanes, Rotaxanes and Knots* (Wiley-VCH, Weinheim, 1999).

[3] Bruns, C. J. & Stoddart, J. F. *The Nature of the Mechanical Bond: From Molecules to Machines* (John Wiley & Sons, Hoboken, NJ, 2017).

[4] Leigh DA, Lusby PJ, Slawin AMZ, Walker DB (2005). Rare and diverse binding modes introduced through mechanical bonding. Angew. Chem. Int. Ed..

[5] Hannam JS (2004). Controlled submolecular translational motion in synthesis: a mechanically interlocking auxiliary. Angew. Chem. Int. Ed..

[6] Tachibana Y, Kawasaki H, Kihara N, Takata T (2006). Sequential *O*- and *N*-acylation protocol for high-yield preparation and modification of rotaxanes: synthesis, functionalization, structure, and intercomponent interaction of rotaxanes. J. Org. Chem..

[7] Cirulli M (2019). Rotaxane-based transition metal complexes: effect of the mechanical bond on structure and electronic properties. J. Am. Chem. Soc..

[8] Berná J (2008). Cadiot-Chodkiewicz active template synthesis of rotaxanes and switchable molecular shuttles with weak intercomponent interactions. Angew. Chem. Int. Ed..

[9] Dichtel WR (2008). Kinetic and thermodynamic approaches for the efficient formation of mechanical bonds. Acc. Chem. Res..

[10] Beves JE, Blight BA, Campbell CJ, Leigh DA, McBurney RT (2011). Strategies and tactics for the metal-directed synthesis of rotaxanes, knots, catenanes and higher order links. Angew. Chem. Int. Ed..

[11] Lewis JEM, Beer PD, Loeb SJ, Goldup SM (2017). Metal ions in the synthesis of interlocked molecules and materials. Chem. Soc. Rev..

[12] Xu Y (2018). A concave-convex π-π template approach enables the synthesis of [10]cycloparaphenylene-fullerene [2]rotaxanes. J. Am. Chem. Soc..

[13] Evans, N. H. Recent advances in the synthesis and application of hydrogen bond templated rotaxanes and catenanes. *Eur. J. Org. Chem*. **2019**, 3320–2243 (2019).

[14] Sauvage J-P (2017). From chemical topology to molecular machines (Nobel Lecture). Angew. Chem. Int. Ed..

[15] Stoddart JF (2017). Mechanically interlocked molecules (MIMs)—molecular shuttles, switches, and machines (Nobel Lecture). Angew. Chem. Int. Ed..

[16] Leigh DA (2016). Genesis of the nanomachines: the 2016 Nobel prize in chemistry. Angew. Chem. Int. Ed..

[17] Kay ER, Leigh DA (2015). Rise of the molecular machines. Angew. Chem. Int. Ed..

[18] Berná J (2005). Macroscopic transport by synthetic molecular machines. Nat. Mater..

[19] Collier CP (1999). Electronically configurable molecular-based logic gates. Science.

[20] Zhu K, O’Keefe CA, Vukotic VN, Schurko RW, Loeb SJ (2015). A molecular shuttle that operates inside a metal–organic framework. Nat. Chem..

[21] Vukotic VN (2015). Mechanically interlocked linkers inside metal–organic frameworks: effect of ring size on rotational dynamics. J. Am. Chem. Soc..

[22] Riss-Yaw B, Morin J, Clavel C, Coutrot F (2017). How secondary and tertiary amide moieties are molecular stations for dibenzo-24-crown-8 in [2]rotaxane molecular shuttles?. Molecules.

[23] Kihara N, Tachibana Y, Kawasaki H, Takata T (2000). Unusually lowered acidity of ammonium group surrounded by crown ether in a rotaxane system and its acylative neutralization. Chem. Lett..

[24] Aucagne V, Hänni KD, Leigh DA, Lusby PJ, Walker DB (2006). Catalytic “click” rotaxanes: a substoichiometric metal-template pathway to mechanically interlocked architectures. J. Am. Chem. Soc..

[25] Crowley JD, Goldup SM, Lee A-L, Leigh DA, McBurney RT (2009). Active metal template synthesis of rotaxanes, catenanes and molecular shuttles. Chem. Soc. Rev..

[26] Denis M, Goldup SM (2017). The active template approach to interlocked molecules. Nat. Rev. Chem..

[27] Hoekman S, Kitching MO, Leigh DA, Papmeyer M, Roke D (2015). Goldberg active template synthesis of a [2]rotaxane ligand for asymmetric transition-metal catalysis. J. Am. Chem. Soc..

[28] Movsisyan LD (2016). Polyyne rotaxanes: stabilization by encapsulation. J. Am. Chem. Soc..

[29] Lewis JEM, Winn J, Cera L, Goldup SM (2016). Iterative synthesis of oligo[n]rotaxanes in excellent yield. J. Am. Chem. Soc..

[30] Brown A, Lang T, Mullen KM, Beer PD (2017). Active metal template synthesis of a neutral indolocarbazole-containing [2]rotaxane host system for selective oxoanion recognition. Org. Biomol. Chem..

[31] Lewis JEM, Modicom F, Goldup SM (2018). Efficient multicomponent active template synthesis of catenanes. J. Am. Chem. Soc..

[32] Jinks MA (2018). Stereoselective synthesis of mechanically planar chiral rotaxanes. Angew. Chem. Int. Ed..

[33] Storey CM, Gyton MR, Andrew RE, Chaplin AB (2018). Terminal alkyne coupling reactions through a ring: mechanistic insights and regiochemical switching. Angew. Chem. Int. Ed..

[34] Franz, M., Januszewski, J. A., Hampel, F. & Tykwinski R. R. [3]Rotaxanes with mixed axles: polyynes and cumulenes. *Eur. J. Org. Chem*. **2019**, 3503–3512 (2019).

[35] Echavarren J (2019). Active template rotaxane synthesis through the Ni-catalyzed cross-coupling of alkylzinc reagents with redox-active esters. Chem. Sci..

[36] De Bo, G, Dolphijn G, McTernan CT, Leigh DA (2017). [2]Rotaxane formation by transition state stabilization. J. Am. Chem. Soc..

[37] Fielden SDP, Leigh DA, McTernan CT, Pérez-Saavedra B, Vitorica-Yrezabal IJ (2018). Spontaneous assembly of rotaxanes from a primary amine, crown ether and electrophile. J. Am. Chem. Soc..

[38] Mock WL, Irra TA, Wepsiec JP, Adhya M (1989). Catalysis by cucurbituril. The significance of bound-substrate destabilization for induced triazole formation. J. Org. Chem..

[39] Hirose K (2007). Highly selective and high-yielding rotaxane synthesis via aminolysis of prerotaxanes consisting of a ring component and a stopper unit. Org. Lett..

[40] Ke C (2013). Pillar[5]arene as a co-factor in templating rotaxane formation. J. Am. Chem. Soc..

[41] Hou X, Ke C, Stoddart JF (2016). Cooperative capture synthesis: yet another playground for copper-free click chemistry. Chem. Soc. Rev..

[42] Orlandini G (2017). Covalent capture of oriented calix[6]arene rotaxanes by a metal-free active template approach. Chem. Commun..

[43] Zanichelli V (2017). Efficient active-template synthesis of calix[6]arene-based oriented pseudorotaxanes and rotaxanes. Org. Biomol. Chem..

[44] Bowers GN, McComb RB, Christensen RC, Schaffer R (1980). High-purity 4-nitrophenol: purification, characterization, and specifications for use as a spectrophotometric reference material. Clin. Chem..

[45] Rüdiger, V., Schneider, H.-J., Solov’ev, V. P., Kazachenko, V. P. & Raevsky, O. A. Crown ether–ammonium complexes: binding mechanisms and solvent effects. *Eur. J. Org. Chem*. **1999**, 1847–1856 (1999).

[46] Birman VB, Li X, Han Z (2007). Nonaromatic amidine derivatives as acylation catalysts. Org. Lett..

[47] Larrivée-Aboussafy C (2010). DBU catalysis of *N*,*N*-carbonyldiimidazole-mediated amidations. Org. Lett..

[48] de Lima EC (2011). DBU as a catalyst for the synthesis of amides via aminolysis of methyl esters. J. Braz. Chem. Soc..

[49] Shawali AS, Harhash A, Sidky MM, Hassaneen HM, Elkaabi SS (1986). Kinetics and mechanism of aminolysis of carbamates. J. Org. Chem..

[50] Koh HJ, Kim OS, Lee HW, Lee I (1997). Kinetics and mechanism of the aminolysis of *p*-nitrophenyl *N*-phenylcarbamates. J. Phys. Org. Chem..

[51] Hogan JC, Gandour RD (1991). Structural requirements for glyme catalysis in butylaminolysis of aryl acetates in chlorobenzene. Identification of -OCH_2_CH_2_OCH_2_CH_2_OCH_2_CH_2_O- as the optimal subunit for catalysis. J. Org. Chem..

[52] Basilio N, García-Río L, Mejuto JC, Pérez-Lorenzo MA (2006). New reaction pathway in the ester aminolysis catalyzed by glymes and crown ethers. J. Org. Chem..

[53] Carpino, L. A., Tsao, J.-H., Ringsdorf, H., Fell, E. & Hettrich, G. The β-(trimethylsilyl)ethoxycarbonyl amino-protecting group. *J. Chem. Soc., Chem. Commun*. **8**, 358−359 (1978).

[54] Castro EA, Ruiz MG, Salinas S, Santos JG (1999). Kinetics and mechanism of the aminolysis of phenyl and 4-nitrophenyl chloroformates in aqueous solution. J. Org. Chem..

[55] Basilio N, García-Río L, Peña-Gallego Á, Pérez-Lorenzo M (2012). Molecular recognition-based catalysis in nucleophilic aromatic substitution: a mechanistic study. New J. Chem..

[56] Gross KC, Seybold PG (2000). Substituent effects on the physical properties and p*K*_a_ of aniline. Int. J. Quantum Chem..

[57] Pedersen CJ (1971). Crystalline complexes of macrocyclic polyethers with thiourea and related compounds. J. Org. Chem..

[58] Elbasyouny A (1983). Host-guest complexes of 18-crown-6 with neutral molecules possessing the structure element XH2 (X = oxygen, nitrogen, or carbon). J. Am. Chem. Soc..

[59] Izatt RM, Pawlak K, Bradshaw JS, Bruening RL (1995). Thermodynamic and kinetic data for macrocycle interaction with cations, anions, and neutral molecules. Chem. Rev..

[60] Barannikov VP, Guseinov SS, V’ugin AI (2002). Molecular complexes of crown ethers in crystals and solutions. Russ. J. Coord. Chem..

[61] Steiner T (2003). C-H···O hydrogen bonding in crystals. Crystallogr. Rev..

[62] Fyfe MCT (1997). Anion-assisted self-assembly. Angew. Chem., Int. Ed. Engl..

[63] Smukste I, Smithrud DB (2003). Structure-function relationship of amino acid-[2]rotaxanes. J. Org. Chem..

[64] Bao X, Isaacsohn I, Drew AF, Smithrud DB (2006). Determining the intracellular transport mechanism of a cleft-[2]rotaxane. J. Am. Chem. Soc..

[65] Hsueh S-Y (2010). Acid/Base- and anion-controllable organogels formed from a urea-based molecular switch. Angew. Chem. Int. Ed..

[66] Ogawa T, Nakazono K, Aoki D, Uchida S, Takata T (2015). Effective approach to cyclic polymer from linear polymer: synthesis and transformation of macromolecular [1]rotaxane. ACS Macro Lett..

[67] Kwan C-S, Chan ASC, Leung KC-F (2016). A fluorescent and switchable rotaxane dual organocatalyst. Org. Lett..

[68] Kolchinski, A. G., Busch, D. H. & Alcock, N. W. Gaining control over molecular threading: benefits of second coordination sites and aqueous–organic interfaces in rotaxane synthesis. *J. Chem. Soc., Chem. Commun*. **12**, 1289−1291 (1995).

[69] Aston PR (1996). Self‐assembling [2]‐ and [3]rotaxanes from secondary dialkylammonium salts and crown ethers. Chem. Eur. J..

[70] Thibeault D, Morin J-F (2010). Recent advances in the synthesis of ammonium-based rotaxanes. Molecules.

[71] Ashton PR (1995). Dialkylammonium ion/crown ether complexes: the forerunners of a new family of interlocked molecules. Angew. Chem., Int. Ed. Engl..

[72] Kosikova T, Hassan NI, Cordes DB, Slawin AMZ, Philp D (2015). Orthogonal recognition processes drive the assembly and replication of a [2]rotaxane. J. Am. Chem. Soc..

[73] Huang Y-L (2007). Using acetate anions to induce translational isomerization in a neutral urea-based molecular switch. Angew. Chem. Int. Ed..

[74] Bandy JA, Truter MR, Vögtle F (1981). The structure of the 1,4,7,10,13,16-hexaoxacyclooctadecane (18-crown-6) bis(dimethyl sulphone) complex. Acta Cryst. B.

[75] Feldblum ES, Arkin IT (2014). Strength of a bifurcated H bond. Proc. Natl Acad. Sci. USA.

[76] Hunter CA (2004). Quantifying intermolecular interactions: guidelines for the molecular recognition toolbox. Angew. Chem. Int. Ed..

[77] McKenzie J, Feeder N, Hunter CA (2016). H-bond competition experiments in solution and the solid state. CrystEngComm.

[78] Laurence C, Berthelot M, Le Questel J-Y, El Ghomari MJ (1995). Hydrogen-bond basicity of thioamides and thioureas. J. Chem. Soc., Perkin Trans..

[79] Caira MR, Mohamed R (1993). Stabilizing role of included solvent in ternary complexation: synthesis, structures and thermal analyses of three 18-crown-6/sulfonamide/acetonitrile inclusion compounds. Acta Cryst. B.

[80] Altieri A (2011). Sulfur-containing amide-based [2]rotaxanes and molecular shuttles. Chem. Sci..

[81] Hamzehee F, Pourayoubi M, Nečas M, Choquesillo-Lazarte D (2017). Extensive analysis of N-H^…^O hydrogen bonding in four classes of phosphorus compounds: a combined experimental and database study. Acta Cryst. C.

[82] Ahmed R (2011). Phosphorus-based functional groups as hydrogen bonding templates for rotaxane formation. J. Am. Chem. Soc..

[83] Metta-Magaña AJ, Pourayoubi M, Pannell KH, Chaijan MR, Eshtiagh-Hosseini H (2012). New organotin(IV)-phosphoramidate complexes: breaking of the PO^…^HN hydrogen bonds and its influence on the molecular packing. J. Mol. Struct..

[84] Colizzi F, Hospital A, Zivanovic S, Orozco M (2019). Predicting the limit of intramolecular hydrogen bonding with classical molecular dynamics. Angew. Chem. Int. Ed..

