# Supplementary material for: Weak functional group interactions revealed through metal-free active template rotaxane synthesis
Source: Nat Commun. 2020 Feb 6;11:744. doi: 10.1038/s41467-020-14576-7 (PMC7005292; doi:10.1038/s41467-020-14576-7)
Supplement: Supplementary file 1 — Supplementary Information [file 41467_2020_14576_MOESM1_ESM.pdf]

Weak Functional Group Interactions Revealed Through Metal-Free Active Template Rotaxane Synthesis

Tian et al.

Department of Chemistry, University of Manchester, Oxford Road, Manchester, M13 9PL, UK

*E-mail: david.leigh@manchester.ac.uk

- Supplementary Information -

1 Contents

1 Contents S2

2 General Methods and Abbreviations S3

3 Optimization of [2]Rotaxane Formation S4

4 Synthesis S6

4.1 Synthesis of Amide Rotaxane **6** S6

4.2 Synthesis of Rotaxane **15** with Carbamate Electrophile S7

4.2.1 Synthesis of Carbamate Electrophile **7** S7

4.2.2 Synthesis of Urea Rotaxane **15** S7

4.3 Synthesis of Urea Rotaxane **16** S8

4.4 Synthesis of Rotaxane **17** with Isothiocyanate Electrophile **9** S9

4.4.1 Synthesis of Isothiocyanate Electrophile **9** S9

4.4.2 Synthesis of Thiourea Rotaxane **17** S9

4.5 Synthesis of Carbamate Rotaxane **18** S10

4.6 Synthesis of Carbamate Rotaxane **19** S10

4.7 Synthesis of Sulfonamide Rotaxane **20** S12

4.8 Synthesis of Phosphoramidate Rotaxane **21** S13

4.9 Synthesis of Phosphinamide Rotaxane **22** S14

4.10 Synthesis of Aniline Rotaxane **24** S15

4.11 Synthesis of Aniline Rotaxane **26** S16

4.12 Synthesis of HOBt ester **S1** S17

5 NMR Spectra of New Compounds S18

6 Single Crystal X-Ray Diffraction S31

7 References S34

Supplementary Methods

2 General Methods and Abbreviations

Unless stated otherwise, reagents were obtained from commercial sources and used without purification. Anhydrous THF (HPLC grade, Fischer Scientific), CH_2Cl_2 (HPLC grade, Fischer Scientific), and toluene (>99%, Fischer Scientific) were obtained by passing the solvent through an activated alumina column on a Phoenix SDS (solvent drying system; JC Meyer Solvent Systems, CA, USA). ^{1}H NMR spectra were recorded on a Bruker Avance III instrument with an Oxford AS600 magnet equipped with a cryoprobe [5mm CPDCH 13C-1H/D] (600 MHz). Chemical shifts are reported in parts per million (ppm) relative to tetramethylsilane from high to low frequency using the residual solvent peak as the internal reference ($CDCl_3$ = 7.26 ppm and CD_3CN = 1.94 ppm). All ^{1}H resonances are reported to the nearest 0.01 ppm. The multiplicity of ^{1}H signals are indicated as: s = singlet; d = doublet; t = triplet; q = quartet; multiplet; br = broad; or combinations of thereof. Coupling constants (*J*) are quoted in Hz and reported to the nearest 0.1 Hz. Where appropriate, averages of the signals from peaks displaying multiplicity were used to calculate the value of the coupling constant. ^{13}C NMR spectra were recorded on the same spectrometer at 298 K with the central resonance of the solvent peak as the internal reference ($CDCl_3$ = 77.16 ppm and CD_3CN = 118.26 ppm). All ^{13}C resonances are reported to the nearest 0.01 ppm. DEPT, COSY, HSQC and HMBC experiments were used to aid structural determination and spectral assignment. Fully characterized compounds were chromatographically homogeneous. Flash column chromatography was carried out using Silica 60 Å (particle size 40 – 63 µm, Sigma Aldrich, UK) as the stationary phase. Preparative TLC was performed using PLC 20 × 20 cm, 60 F_{254} preparatory plates of various thicknesses (250 – 2000 µm). Analytical TLC was performed on precoated silica gel plates (0.25 mm thick, 60 F254, Merck, Germany) and visualized using both short and long waved ultraviolet light in combination with standard laboratory stains (acidic potassium permanganate, iodine vapor). Low resolution ESI mass spectrometry was performed with a Thermo Scientific LCQ Fleet Ion Trap Mass Spectrometer or an Agilent Technologies 1200 LC system with an Advion Expression CMS L single quadrupole MS detector. High-resolution mass spectrometry was carried out at the Mass Spectrometry Service, School of Chemistry, The University of Manchester. Synthetic protocols and characterization data for compounds **2**, **3, 4** and **S2** were reported previously.[1,2] Other compounds synthesized according to literature procedures are detailed in the relevant sections.

Abbreviations: ASAP: atmospheric solids analysis probe; DBU: 1,8-diazabicyclo(5.4.0)undec-7-ene; EDC: *N*-(3-dimethylaminopropyl)-*N'*-ethylcarbodiimide hydrochloride; eq: equivalents; ESI: electrospray ionization; Et_3N: triethylamine; EtOAc: ethyl acetate; h: hours; HOBt: 1-hydroxybenzotriazole; HRMS: high resolution mass spectrometry; MeCN: acetonitrile; MeOH: methanol; min: minutes; PE: petroleum ether (boiling point 40-60 °C); PMP: 1,2,2,6,6-pentamethylpiperidine rt: room temperature; t-bu: tertiary butyl; THF: tetrahydrofuran.

3 Optimization of [2]Rotaxane Formation

Supplementary Table 1: Screening of amines for *N*-Acylation.[a]

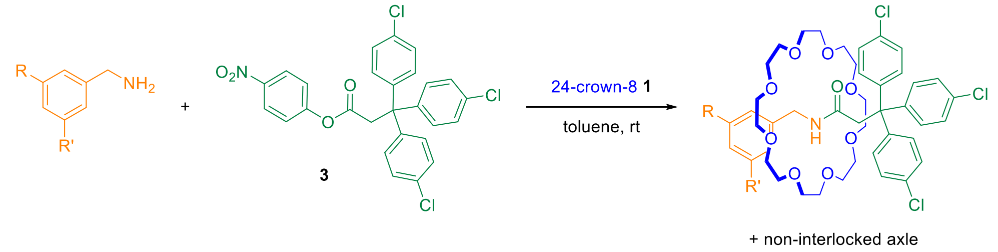

Entry	R	R'	% Rotaxane after 1 h[b]	% Rotaxane after 24 h[b]	Rotaxane:non-interlocked axle after 24 h[b]
1	t-Bu	t-Bu	56	58	8:1
2	Me	Me	29	57	2:1
3	OMe	OMe	44	65	6:1
4	Me	CF_3	46	75	35:1
5	CF_3	CF_3	61	84	>100:1

[a]Conditions: 1 eq. of amine, **3** and **1** (0.14 M), toluene, rt; [b]Determined by ^{1}H NMR analysis;

Supplementary Table 2: Screening of bases for *N*-Acylation.[a]

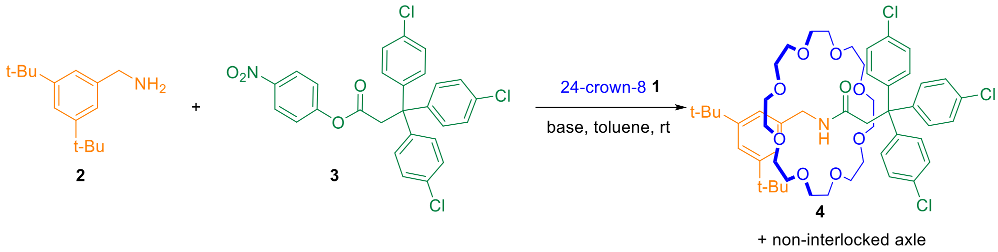

Entry	Base	% Rotaxane after 1 h[b]	% Rotaxane after 24 h[b]	Rotaxane:non-interlocked axle after 24 h[b]
1	none	56	58	8:1
2	10 eq. Et_3N	68	92	17:1
3	10 eq. PMP[c]	61	85	12:1
4	10 eq. DBU	10	n/a	1:9
5	10 eq. 2,6-lutidine	53	81	14:1

[a]Conditions: 1 eq. of **1**, **2** and **3** (0.14 M), base, toluene, rt; [b]Determined by ^{1}H NMR analysis; [c]PMP: 1,2,2,6,6-pentamethylpiperidine

Supplementary Table 3: Screening of electrophiles for *N*-Acylation

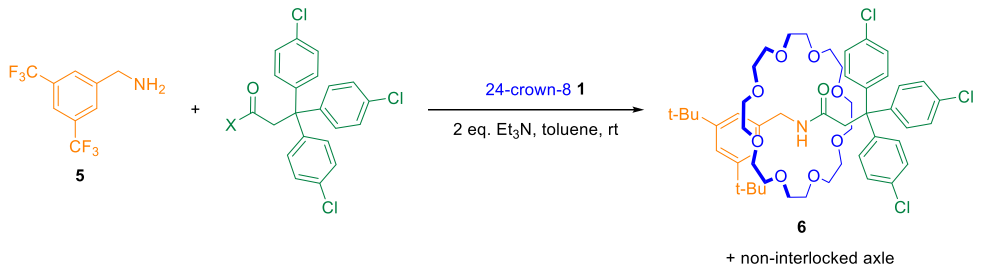

Entry	X	% Rotaxane after 1 h[b]	Rotaxane:non-interlocked axle after 1 h[b]
1	1-hydroxybenzotriazole (**S1**)	40	0.9:1
2	Cl (**S2**)	54	1.4:1

[a]Conditions: 1 eq. of **1**, **5** and electrophile (0.14 M), 2 eq. Et_3N, toluene, rt; [b]Determined by ^1H NMR analysis; no change observed 1 hour.

4 Synthesis

4.1 Synthesis of Amide Rotaxane **6**

Amine **5** is commercially available.

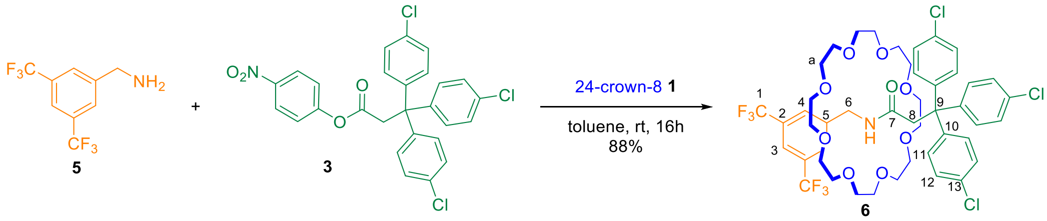

To a stirred solution of **1** (49 mg, 0.14 mmol, 1.0 eq.) and **5** (34 mg, 0.14 mmol, 1.0 eq.) in toluene (1.0 mL) at room temperature was added **3** (73 mg, 0.14 mmol, 1.0 eq.). The mixture was stirred at room temperature overnight and concentrated under reduced pressure. Flash chromatography of the crude residue (SiO_2, EtOAc/PE 1:4) afforded **6** (120 mg, 0.12 mmol, 88 %) as a colorless solid.

^{1}H NMR (600 MHz, $CDCl_3$, 295 K) δ 8.55 (d, *J* = 1.7 Hz, 2H, H_4), 7.68 (s, 1H, H_3), 7.19 (m, 12H, $H_{11,12}$), 7.05 (t, *J* = 4.4 Hz, 1H, N-H), 4.55 (d, *J* = 4.3 Hz, 2H, H_6), 3.49 (s, 2H, H_8), 3.42 – 3.36 (m, 16H, H_a), 3.21 – 3.15 (m, 16H, $H_{a'}$).

^{13}C NMR (151 MHz, $CDCl_3$, 295 K) δ 168.02 (C_7), 145.93 (C_{10}), 142.59 (C_5), 134.10 (C_4), 131.97 (C_{13}), 130.74 (C_{11}), 129.27 (q, *J* = 32.4 Hz, C_2), 127.94 (C_{12}), 124.23 (q, *J* = 272.4 Hz, C_1), 119.52 (q, *J* = 4.5 Hz, C_3), 70.65 (C_a), 54.17 (C_9), 46.78 (C_8), 43.02 (C_6).

HRMS (ESI^+): m/z = 982.2669 $[M+H]^+$, calculated for $C_{46}H_{53}O_9NCl_3F_6$ = 982.2685.

4.2 Synthesis of Rotaxane **15** with Carbamate Electrophile

4.2.1 Synthesis of Carbamate Electrophile **7**

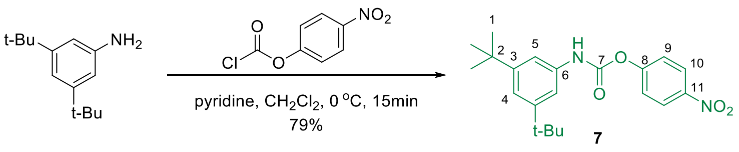

To a stirring solution of 3,5-di-tert-butylaniline (205 mg, 1.0 mmol, 1.0 eq.) and pyridine (0.089 mL, 1.1 mmol, 1.1 eq.) in CH_2Cl_2 (4 mL) at 0 °C was added 4-nitrophenyl chloroformate (0.138 mL, 1.1 mmol, 1.1 eq.) dropwise over 5 mins. The resulting mixture was stirred at 0 °C for 10 min and then washed with 5% aqueous citric acid. The organic fraction was dried over anhydrous $MgSO_4$ and concentrated under reduced pressure. Flash chromatography of the crude residue (SiO_2, EtOAc/PE 1:10) afforded **7** as a colorless solid (293 mg, 0.79 mmol, 79%).

^{1}H NMR (600 MHz, $CDCl_3$, 295 K) δ 8.29 (d, *J* = 9.1 Hz, 2 H, H_{10}), 7.40 (d, *J* = 9.1 Hz, 2H, H_9), 7.31 (s, 2H, H_5), 7.22 (s, 1H, H_4), 6.99 (s, 1H, N-H), 1.33 (s, 1H, H_1).

^{13}C NMR (151 MHz, $CDCl_3$, 295K) δ 155.61 (C_7), 152.24 (C_3), 150.45 (C_{11}), 145.15 (C_8), 136.09 (C_6), 125.36 (C_{10}), 122.37 (C_9), 118.88 (C_4), 113.79 (C_5), 35.14 (C_2), 31.51 (C_1).

HRMS (ESI$^+$): m/z = 371.1962 $[M+H]^+$, calculated for $C_{21}H_{27}O_4N_2$ = 371.1965.

4.2.2 Synthesis of Urea Rotaxane **15**

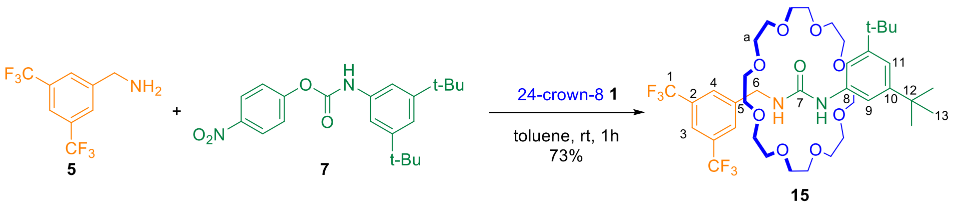

To a stirring solution of **1** (49 mg, 0.14 mmol, 1.0 eq.) and **5** (34 mg, 0.14 mmol, 1.0 eq.) in toluene (1.0 mL) was added **7** (52 mg, 0.14 mmol, 1.0 eq.). The mixture was stirred for one hour and then concentrated under reduced pressure. Flash chromatography of the crude residue (SiO_2, EtOAc/PE 1:5 then CH_2Cl_2/MeOH 50:1) afforded rotaxane **15** (85 mg, 0.10 mmol, 73 %) as a colorless solid.

^{1}H NMR (600 MHz, $CDCl_3$, 295 K) δ 8.53 (s, 2H, H_4), 7.70 (s, 1H, H_3), 7.33 (s, 2H, H_9), 7.29 (s, 1H, N-$H_{aniline}$), 6.99 (s, 1H, H_{11}), 6.04 (t, *J* = 4.9 Hz, 1H, N-$H_{benzylic}$), 4.83 (d, *J* = 4.9 Hz, 2H, H_6), 3.58 – 3.52 (m, 16H, H_a), 3.34 – 3.29 (m, 16H, $H_{a'}$), 1.31 (s, 18H, H_{13}).

^{13}C NMR (151 MHz, $CDCl_3$, 295 K) δ 155.96 (C_7), 150.92 (C_{10}), 143.77 (C_5), 140.45 (C_8), 132.16 (C_4), 129.73 (q, *J* = 32.5 Hz, C_2), 124.18 (q, *J* = 272.5 Hz, C_1), 119.53 (q, *J* = 4.5 Hz) (C_3), 115.23 (C_{11}), 113.32 (C_9), 70.70 (C_a), 43.97 (C_6), 34.98 (C_{12}), 31.65 (C_{13}).

HRMS (ESI$^+$): m/z = 827.4253 $[M+H]^+$, calculated for $C_{40}H_{61}O_9N_2F_6$ = 827.4276.

4.3 Synthesis of Urea Rotaxane **16**

3,5-Dimethylphenyl isocyanate **8** is commercially available.

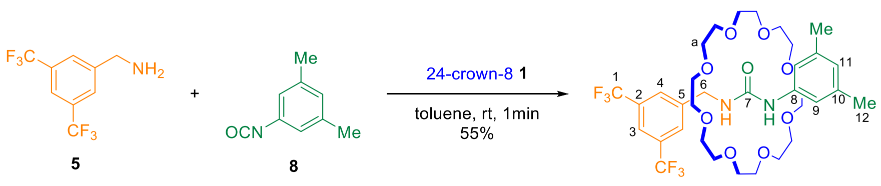

5 8 16

To a stirring solution of **5** (13.8 mg, 57 µmol, 1 eq.) and **1** (20.0 mg, 57 µmol, 1 eq.) in toluene (400 µl) was added **8** (12.4 mg, 84 µmol, 1.5 eq.). The resulting solution was stirred for one minute, after which full conversion of **5** was observed. Preparative thin layer chromatography of the reaction mixture (SiO_2, EtOAc/PE 3:7, 1 elution then EtOAc/PE 1:1, 1 elution) followed by size exclusion chromatography (SX3 beads, CH_2Cl_2) afforded **16** (22.8 mg, 31 µmol, 55 %) as a colorless solid.

^{1}H NMR (600 MHz, $CDCl_3$, 295 K) δ 8.54 (s, 2H, H_4), 7.70 (s, 1H, H_3), 7.23 (s, 1H, N-$H_{aniline}$), 7.05 (s, 2H, H_9), 6.56 (s, 1H, H_{11}), 6.07 (t, J = 4.8 Hz, 1H, N-$H_{benzylic}$), 4.80 (d, J = 4.7 Hz, 2H, H_6), 3.55 (m, 16H, H_a), 3.39 – 3.29 (m, 16H, $H_{a'}$), 2.26 (s, 6H, H_{12}).

^{13}C NMR (151 MHz, $CDCl_3$, 295 K) δ 155.70 (C_7), 143.73 (C_5), 140.97 (C_8), 138.33 (C_{10}), 132.28 (C_4), 129.70 (q, J = 32.5 Hz, C_2), 125.10 (q, J = 272.4 Hz, C_1), 122.92 (C_{11}), 119.63 – 119.44 (m, C_3), 115.91 (C_9), 70.74 (C_a), 43.73 (C_6), 21.67 (C_{12}).

HRMS (ESI^+): m/z = 765.3156 $[M+Na]^+$, calculated for $C_{34}H_{48}O_9N_2F_6Na$ = 765.3156.

4.4 Synthesis of Rotaxane **17** with Isothiocyanate Electrophile **9**

4.4.1 Synthesis of Isothiocyanate Electrophile **9**

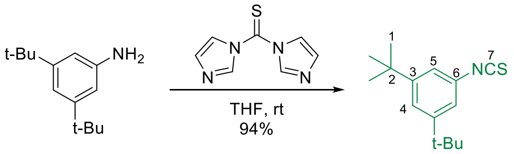

9

To a solution of 3,5-di-tert-butylaniline (205 mg, 1.0 mmol, 1.0 eq.) in THF (5 ml) at room temperature was added 1,1'-thiocarbonyldiimidazole (196 mg, 1.1 mmol, 1.1 eq.) and the resulting solution was stirred overnight. The resulting mixture was concentrated under reduced pressure. Flash chromatography of the crude residue (PE) afforded **9** (233 mg, 0.94 mmol, 94 %) as a colorless solid.

^{1}H NMR (600 MHz, $CDCl_3$, 295 K) δ 7.33 (t, J = 1.8 Hz, 1H, H_4), 7.06 (d, J = 1.8 Hz, 2H, H_5), 1.31 (s, 18H, H_1).

^{13}C NMR (151 MHz, $CDCl_3$, 295 K) δ 152.64 (C_3), 133.32 (C_7), 130.41 (C_6), 121.84 (C_4), 120.21 (C_5), 35.13 (C_2), 31.37 (C_1).

Analytical data matches with literature values.[3]

4.4.2 Synthesis of Thiourea Rotaxane **17**

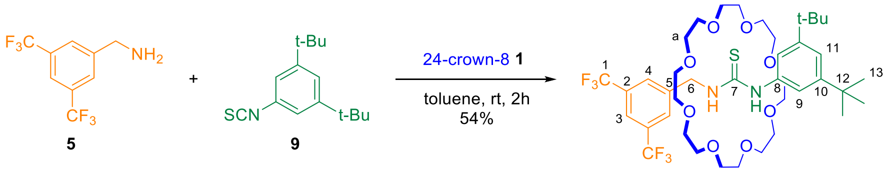

17

To a vial containing **5** (13.8 mg, 57 µmol, 1 eq.) and **9** (14.0 mg, 57 µmol, 1 eq.) was added **1** (20.0 mg, 57 µmol, 1 eq.) in toluene (400 µl). The resulting solution was left for 2.5 h. Preparative thin layer chromatography of the reaction mixture (SiO_2, EtOAc/PE 3:7, 1 elution) afforded **17** (26.0 mg, 31 µmol, 54 %) as a colorless solid.

Significant broadening of peaks in the ^{1}H and ^{13}C NMR spectra occur at 295 K in $CDCl_3$.

^{1}H NMR (600 MHz, $CDCl_3$, 325 K) δ 8.57 (brs, 2H, H_4), 8.15 (brs, 1H, $N_{benzylic}$-H), 7.75 (s, 1H, H_3), 7.53 (m, 3H, H_9, $N_{aniline}$-H), 7.12 (s, 1H, H_{11}), 5.03 (d, J = 4.3 Hz, 2H, H_6), 3.65 – 3.57 (m, 16H, H_a), 3.40 – 3.29 (m, 16H, $H_{a'}$), 1.33 (s, 18H, H_{13}).

^{13}C NMR (151 MHz, $CDCl_3$, 325 K) δ 179.96 (C_7), 150.61 (C_{10}), 140.12 (C_8), 133.12 (C_4), 130.09 (q, J = 31.4 Hz, C_2), 124.23 (q, J = 272.1 Hz, C_1), 120.22 (C_3), 117.72 ($C_{9,11}$), 70.95(C_a), 47.81 (C_6), 35.09 (C_{12}), 31.71 (C_{13}). C_9 and C_{11} could not be resolved. C_5 not observed.

HRMS (ESI^+): m/z = 843.4042 $[M+H]^+$, calculated for $C_{40}H_{61}O_8N_2F_6S$ = 843.4047.

4.5 Synthesis of Carbamate Rotaxane **18**

4-Nitrophenyl 2-(trimethylsilyl)ethyl carbonate **10** is commercially available.

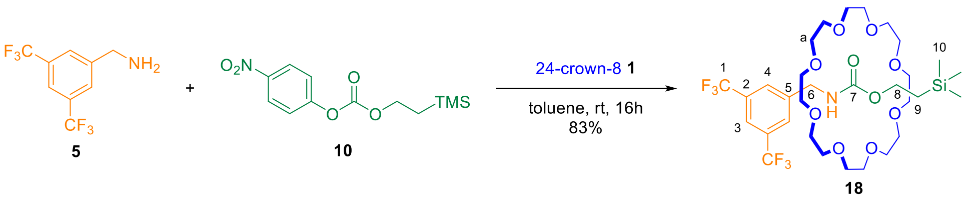

To a stirring solution of 24-crown-8 **1** (49 mg, 0.14 mmol, 1.0 eq.) and amine **5** (34 mg, 0.14 mmol, 1.0 eq.) in toluene (1.0 mL) was added **10** (40 mg, 0.14 mmol, 1.0 eq.). The mixture was stirred for 16 hours and then concentrated under reduced pressure. Flash chromatography of the crude residue (SiO_2, EtOAc/PE 1:3 then CH_2Cl_2/MeOH 30:1) afforded **18** (86 mg, 0.12 mmol, 83 %) as a colorless solid.

A minor conformer of **18** (in a *ca.* 1:5 ratio) exists in $CDCl_3$ at 295 K. Assignments are given for the major conformer.

^{1}H NMR (600 MHz, $CDCl_3$, 295 K) δ 8.58 (s, 2H, H_4), 7.66 (s, 1H, H_3), 6.61 (t, *J* = 4.4 Hz, 1H, N-H), 4.71 (d, *J* = 4.3 Hz, 2H, H_6), 4.16-4.11 (m, 2H, H_8), 3.53 – 3.47 (m, 16H, H_a), 3.26-3.20 (m, 16H, $H_{a'}$), 0.99 – 0.93 (m, 2H, H_9), 0.01 (s, 9H, H_{10}).

^{13}C NMR (151 MHz, $CDCl_3$, 295 K) δ 156.93 (C_7), 142.18 (C_5), 133.35 (q, *J* = 3.7 Hz, C_4), 129.13 (q, *J* = 32.5 Hz, C_2), 124.28 (q, *J* = 272.5 Hz, C_1), 119.40 (q, *J* = 4.5 Hz, C_3), 70.66 (C_a), 61.93 (C_8), 44.51 (C_6), 18.14 (C_9), -1.28 (C_{10}).

HRMS (ESI^+): m/z = 740.3250 $[M+H]^+$, calculated for $C_{31}H_{52}O_{10}NF_6Si$ = 740.3259.

4.6 Synthesis of Carbamate Rotaxane **19**

9-Fluorenylmethyl chloroformate (Fmoc-Cl) **11** is commercially available.

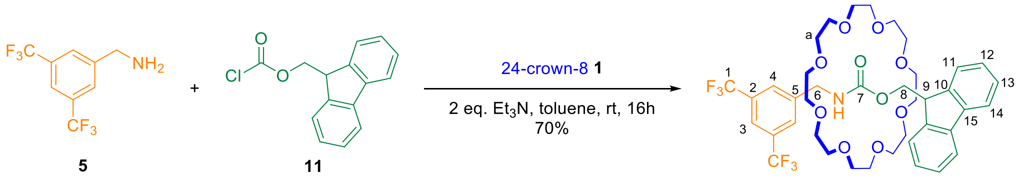

To a stirring solution of **1** (49 mg, 0.14 mmol, 1.0 eq.) and **5** (34 mg, 0.14 mmol, 1.0 eq.) in toluene (1.0 mL) was added **11** (36 mg, 0.14 mmol, 1.0 eq.) and Et_3N (39 µL, 0.28 mmol, 2.0 eq.). The mixture was stirred for 16 hours and then concentrated under reduced pressure. Flash chromatography of the crude residue (SiO_2, CH_2Cl_2/MeOH 30:1) afforded **19** (79 mg, 0.10 mmol, 70%) as a colorless solid.

A minor conformer of **19** (in a *ca.* 1:8 ratio) exists in $CDCl_3$ at 295 K. Assignments are given for the major conformer.

^{1}H NMR (600 MHz, $CDCl_3$, 295 K) δ 8.54 (s, 2H, H_4), 7.73 (d, *J* = 6.0 Hz, 2H, H_{14}), 7.71 (d, *J* = 6.0 Hz, 2H, H_{11}), 7.67 (s, 1H, H_3), 7.36 (t, *J* = 7.4 Hz, 2H, H_{13}), 7.27 (t, *J* = 7.4 Hz, 2H, H_{12}), 6.74 (t, *J* = 4.2 Hz, 1H, N-H), 4.70 (d, *J* = 4.2 Hz, 2H, H_6), 4.54 (d, *J* = 6.1 Hz, 2H, H_8), 4.17 (t, *J* = 6.1 Hz, 1H, H_9), 3.43-3.37 (m, 16H, H_a), 3.21-3.16 (m, 16H, $H_{a'}$).

^{13}C NMR (151 MHz, $CDCl_3$, 295 K) δ 156.39 (C_7), 144.66 (C_{10}), 141.72 (C_5), 141.54 (C_{15}), 133.44 (q, *J* = 3.7 Hz, C_4), 129.14 (q, *J* = 32.5 Hz, C_2), 127.47 (C_{13}), 127.01 (C_{12}), 125.28 (C_{11}), 124.24 (q, *J* = 272.5 Hz, C_1), 119.79 (C_{14}), 119.48 (q, *J* = 3.8 Hz, C_3), 70.61 (C_a), 65.09 (C_8), 47.73 (C_9), 44.57 (C_6).

HRMS (ESI^+): m/z = 818.3310 $[M+H]^+$, calculated for $C_{40}H_{50}O_{10}NF_6$ = 818.3333.

4.7 Synthesis of Sulfonamide Rotaxane **20**

3,5-Di-tert-butylbenzenesulfonyl chloride **12** was synthesized from 1,3,5-tri-tert-butylbenzene according to a literature procedure.[4]

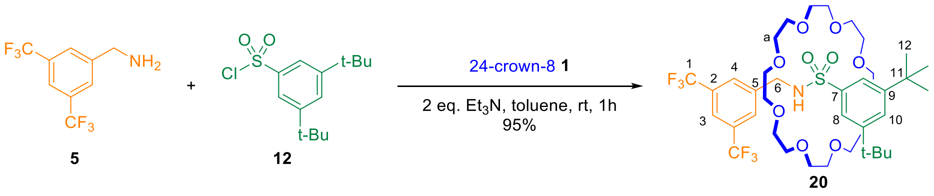

To a stirring solution of **1** (49 mg, 0.14 mmol, 1.0 eq.) and **5** (34 mg, 0.14 mmol, 1.0 eq.) in toluene (1.0 mL) at room temperature was added **12** (40 mg, 0.14 mmol, 1.0 eq.) and Et_3N (39 µL, 0.28 mmol, 2.0 eq.). The mixture was stirred for one hour and then concentrated under reduced pressure. Flash chromatography of the crude residue (SiO_2, CH_2Cl_2/MeOH 30:1) afforded **20** (113 mg, 0.13 mmol, 95 %) as a colorless solid.

^{1}H NMR (600 MHz, $CDCl_3$, 295 K) δ 8.60 (s, 2H, H_4), 7.78 (d, *J* = 1.7 Hz, 2H, H_8), 7.67 (s, 1H, H_3), 7.60 (s, 1H, H_{10}), 6.50 (t, *J* = 5.7 Hz, 1H, N-H), 4.55 (d, *J* = 5.7 Hz, 2H, H_6), 3.45 – 3.40 (m, 16H, H_a), 3.23 – 3.18 (m, 16H, $H_{a'}$), 1.35 (s, 18H, H_{12}).

^{13}C NMR (151 MHz, $CDCl_3$, 295 K) δ 151.53 (C_9), 140.83 (C_5), 140.56 (C_7), 132.60 (q, *J* = 3.0 Hz, C_4), 129.25 (q, *J* = 32.6 Hz, C_2), 126.72 (C_{10}), 124.24 (q, *J* = 272.5 Hz, C_1), 121.83 (C_8), 119.65 (q, *J* = 4.5 Hz, C_3), 70.72 (C_a), 45.98 (C_6), 35.26 (C_{11}), 31.48 (C_{12}).

HRMS (ESI$^+$): m/z = 848.3824 $[M+H]^+$, calculated for $C_{39}H_{60}O_{10}NF_6S$ = 848.3837.

4.8 Synthesis of Phosphoramidate Rotaxane **21**

Diphenyl phosphoryl chloride **13** is commercially available.

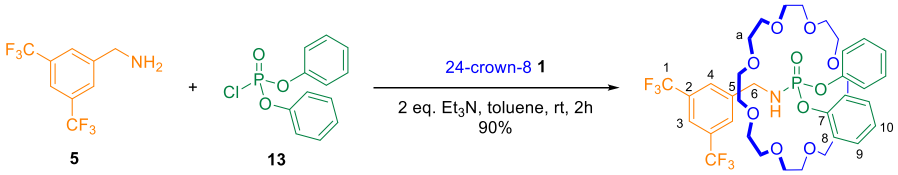

5 **13** **21**

To a vigorously stirring solution of **5** (13.8 mg, 57 µmol, 1 eq.), Et_3N (16 µl, 114 µmol, 2 eq.) and **1** (20.0 mg, 57 µmol, 1 eq.) in toluene (400 µl) was added **13** (12 µl, 57 µmol, 1 eq.) dropwise. The resulting solution was left to stir for two hours, during which time gelation occurred. Preparative thin layer chromatography of the reaction mixture (SiO_2, acetone/PE 1:4, 1 elution) afforded **21** (42.1 mg, 51 µmol, 90 %) as a colorless solid.

^{1}H NMR (600 MHz, $CDCl_3$, 295 K) δ 8.66 (s, 2H, H_4), 7.69 (s, 1H, H_3), 7.37 – 7.32 (m, 4H, H_8), 7.31 – 7.25 (m, 4H, H_9), 7.10 (t, J = 7.3 Hz, 2H, H_{10}), 5.34 (dt, J = 11.2, 5.3 Hz, 1H, N-H), 4.86 (t, J = 4.3 Hz, 2H, H_6), 3.57 – 3.45 (m, 16H, H_a), 3.33 – 3.19 (m, 16H, $H_{a'}$).

^{13}C NMR (151 MHz, $CDCl_3$, 295 K) δ 151.67 (d, J = 7.1 Hz, C_7), 142.77 (d, J = 15.9 Hz, C_5), 131.84 (br, C_4), 129.46 (C_9), 129.38 (q, J = 32.6 Hz, C_2), 125.24 (q, J = 272.4 Hz, C_1), 124.38 (C_{10}), 121.05 (d, J = 4.8 Hz, C_8), 119.27 (q, J = 4.2 Hz, C_3), 70.79 (C_a), 44.50 (C_6).

HRMS (ESI$^+$): m/z = 850.2742 $[M+Na]^+$, calculated for $C_{37}H_{48}O_{11}NF_6PNa$ = 850.2761.

4.9 Synthesis of Phosphinamide Rotaxane **22**

Diphenylphosphinic chloride **20** is commercially available.

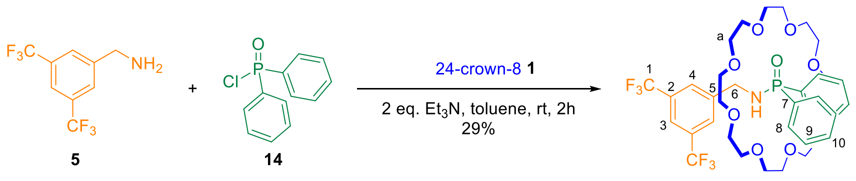

To a vigorously stirring solution of **5** (13.8 mg, 57 µmol, 1 eq.), Et_3N (16 µl, 114 µmol, 2 eq.) and **1** (20.0 mg, 57 µmol, 1 eq.) in toluene (400 µl) was added **14** (11 µl, 57 µmol, 1 eq.) dropwise. Immediate gelation occurred. The resulting mixture was left to stir for 2 h. Preparative thin layer chromatography of the reaction mixture (SiO_2, EtOAc/PE 4:1, 2 elutions) afforded **22** (13.2 mg, 17 µmol, 29 %) as a colorless solid.

^{1}H NMR (600 MHz, $CDCl_3$, 295 K) δ 8.73 (s, 2H, H_4), 8.09 – 7.98 (m, 4H, H_9), 7.67 (s, 1H, H_3), 7.49 – 7.37 (m, 6H, $H_{8,10}$), 4.86 (brs, 1H, N-H), 4.52 (t, *J* = 4.9 Hz, 2H, H_6), 3.44 – 3.33 (m, 16H, H_a), 3.21 – 3.08 (m, 16H, $H_{a'}$).

^{13}C NMR (151 MHz, $CDCl_3$, 295 K) δ 142.92 (d, *J* = 13.0 Hz, C_5), 134.34 (C_7), 133.28 (C_4), 133.02 (d, *J* = 9.7 Hz, C_9), 131.02 (d, *J* = 2.7 Hz, C_{10}), 129.04 (q, *J* = 32.4 Hz, C_2), 128.11 (d, *J* = 12.3 Hz, C_8), 124.39 (q, *J* = 272.6 Hz, C_1), 119.71 – 119.29 (m, C_3), 70.68 (C_a), 43.72 (C_6).

HRMS (ESI^+): m/z = 818.2854 $[M+Na]^+$, calculated for $C_{37}H_{48}O_9NF_6PNa$ = 818.2863.

4.10 Synthesis of Aniline Rotaxane **24**

5-Fluoro-2-nitrobenzotrifluoride **23** is commercially available.

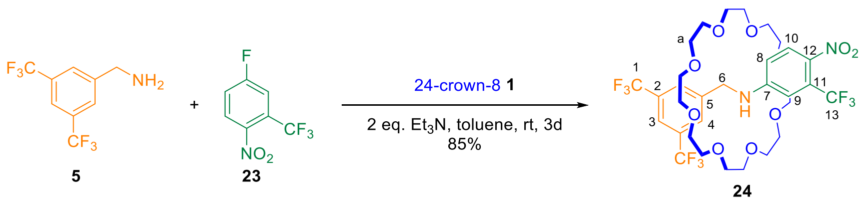

To a stirring solution of **5** (13.8 mg, 57 µmol, 1 eq.), Et_3N (16 µl, 114 µmol, 2 eq.) and **1** (20.0 mg, 57 µmol, 1 eq.) in toluene (400 µl) was added **23** (17.6 mg, 86 µmol, 1.5 eq.). The resulting mixture was left to stir for 3 days. Flash chromatography of the crude residue (SiO_2, EtOAc/PE 1:9 to 1:3) afforded **24** (38.1 mg, 49 µmol, 85 %) as a yellow solid.

^{1}H NMR (600 MHz, $CDCl_3$, 295 K) δ 8.47 (s, 2H, H_4), 8.09 (d, *J* = 9.1 Hz, 1H, H_{10}), 7.77 (s, 1H, H_3), 7.11 (s, 1H, H_9), 6.81 (d, *J* = 9.1 Hz, 1H, H_8), 6.72 (t, *J* = 4.3 Hz, 1H, N-H), 4.80 (d, *J* = 4.3 Hz, 2H, H_6), 3.41 – 3.37 (m, 16H, H_a), 3.31 – 3.25 (m, 16H, $H_{a'}$).

^{13}C NMR (151 MHz, $CDCl_3$, 295 K) δ 153.28 (C_7), 141.68 (C_5), 133.65 (C_{12}), 132.83 (C_4), 131.47 ($C_{8/9}$), 130.15 (q, *J* = 32.8 Hz, C_2), 129.64 (brs, C_{10}), 128.59 ($C_{8/9}$), 126.61 – 126.03 (brm, C_{11}), 123.99 (q, *J* = 272.4 Hz, C_1), 122.94 (q, *J* = 273.3 Hz, C_{13}), 120.51 – 120.25 (m, C_3), 70.81 (C_a), 46.34 (C_6).

HRMS (ESI^+): m/z = 785.2662 $[M+H]^+$, calculated for $C_{32}H_{42}F_9N_2O_{10}$ = 785.2690.

4.11 Synthesis of Aniline Rotaxane **26**

1-Chloro-2,4-dinitrobenzene **25** is commercially available.

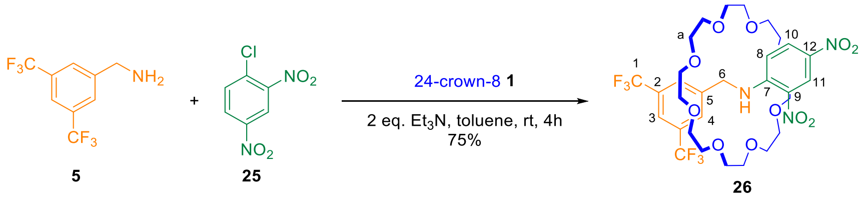

To a stirring solution of **5** (13.8 mg, 57 µmol, 1 eq.), Et_3N (16 µl, 114 µmol, 2 eq.) and **1** (20.0 mg, 57 µmol, 1 eq.) in toluene (400 µl) was added **25** (11.3 mg, 57 µmol, 1 eq.). The resulting mixture was left to stir for 4 h. Flash chromatography of the crude residue (SiO_2, EtOAc/PE 1:9 to 1:3) afforded **26** (32.7 mg, 43 µmol, 75 %) as a yellow solid.

^{1}H NMR (600 MHz, $CDCl_3$, 295 K) δ 9.01 (d, *J* = 2.7 Hz, 1H, H_{11}), 8.68 (t, *J* = 5.1 Hz, 1H, N-H), 8.44 (s, 2H, H_4), 8.19 (dd, *J* = 9.7, 2.7 Hz, 1H, H_{10}), 7.74 (s, 1H, H_3), 7.58 (d, *J* = 9.7 Hz, 1H, H_8), 5.12 (d, *J* = 5.1 Hz, 2H, H_6), 3.37 – 3.32 (m, 16H, H_a), 3.27 – 3.21 (m, 16H, $H_{a'}$).

^{13}C NMR (151 MHz, $CDCl_3$, 295 K) δ 148.32 (C_7), 140.31 (C_5), 134.35 ($C_{9/12}$), 133.62 – 133.08 (m, C_4), 131.10 ($C_{9/12}$), 129.72 (q, *J* = 32.7 Hz, C_2), 128.91 (C_{10}), 124.10 (q, *J* = 273.3, C_1), 123.94 (C_{11}), 120.69 – 120.39 (m, C_3), 117.14 (C_8), 70.79 (C_a), 47.47 (C_6).

HRMS (ESI^-): m/z = 760.2513 $[M-H]^-$, calculated for $C_{31}H_{40}F_6N_3O_{12}$ = 760.2522.

4.12 Synthesis of HOBt ester **S1**

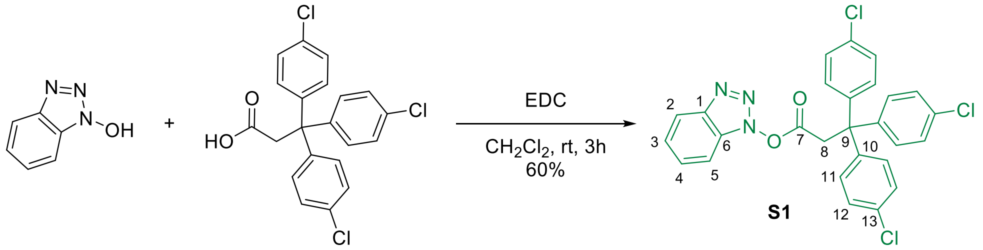

A solution of 3,3,3-tris(4-chlorophenyl)propionic acid (406 mg, 1.0 mmol, 1.0 eq.), 1-hydroxybenzotriazole hydrate (236 mg, 2.0 mmol, 2.0 eq.) and *N*-(3-dimethylaminopropyl)-*N′*-ethylcarbodiimide hydrochloride (345 mg, 1.8 mmol, 1.8 eq.) in CH_2Cl_2 (20 ml) was stirred at room temperature for 3 hours. Flash chromatography of the crude residue (SiO_2, EtOAc/PE 1:9) afforded **S1** as a colorless oil (313 mg, 0.60 mmol, 60%).

^{1}H NMR (600 MHz, $CDCl_3$, 295 K) δ 7.98 (d, *J* = 8.4 Hz, 1H, $H_{2/5}$), 7.47-7.44 (m, 1H, $H_{3/4}$), 7.39 – 7.30 (overlapped, 7H, $H_{3/4,11}$), 7.22 – 7.17 (m, 6H, H_{12}), 6.65 (m, 1H, $H_{2/5}$), 4.12 (s, 2H, H_8).

^{13}C NMR (151 MHz, $CDCl_3$, 295 K) δ 166.62 (C_7), 143.33 ($C_{1/6}$), 143.11 (C_{10}), 133.50 (C_{13}), 130.33 (C_{12}), 128.89 ($C_{3/4}$), 128.83 (C_{11}), 128.39 ($C_{1/6}$), 124.96 ($C_{3/4}$), 120.51 ($C_{2/5}$), 107.99 ($C_{2/5}$), 54.92 (C_9), 43.20 (C_8).

HRMS (ASAP$^+$): m/z = 522.0536 $[M+H]^+$, calculated for $C_{27}H_{19}O_2N_3Cl_3$ = 522.0537.

5 NMR Spectra of New Compounds

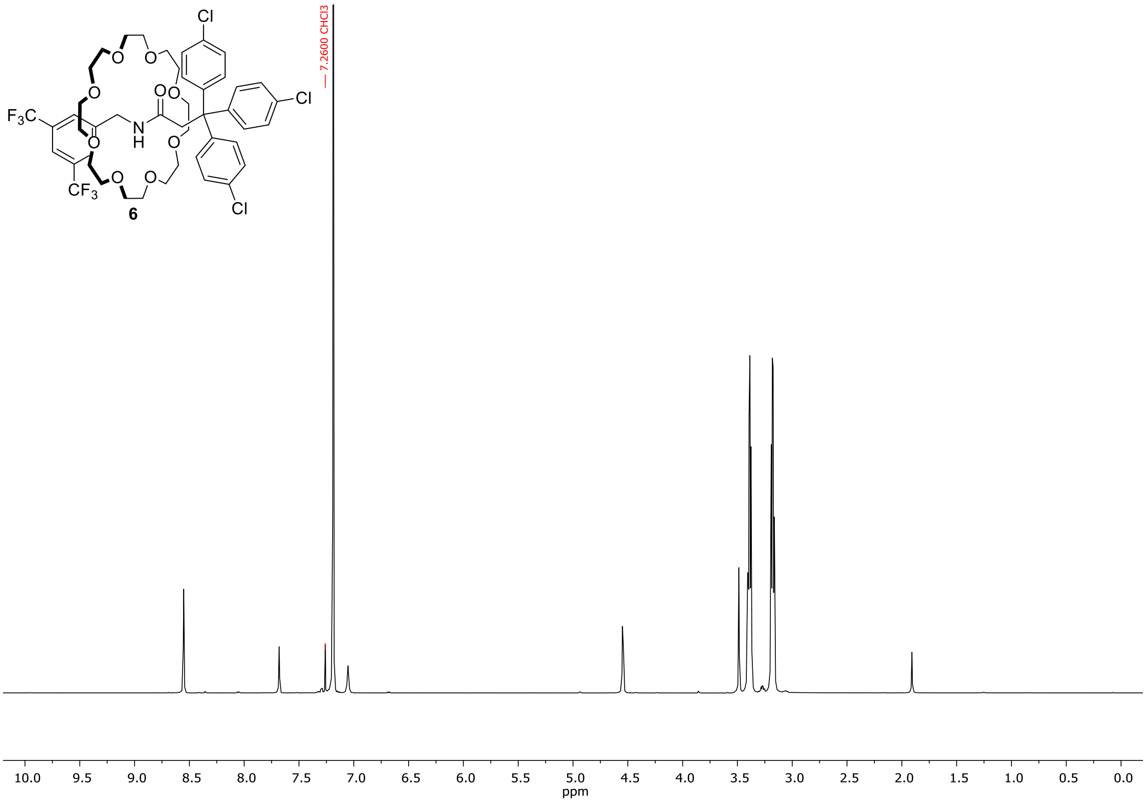

Supplementary Figure 1: ^{1}H NMR ($CDCl_3$, 600 MHz, 295 K) of **6**.

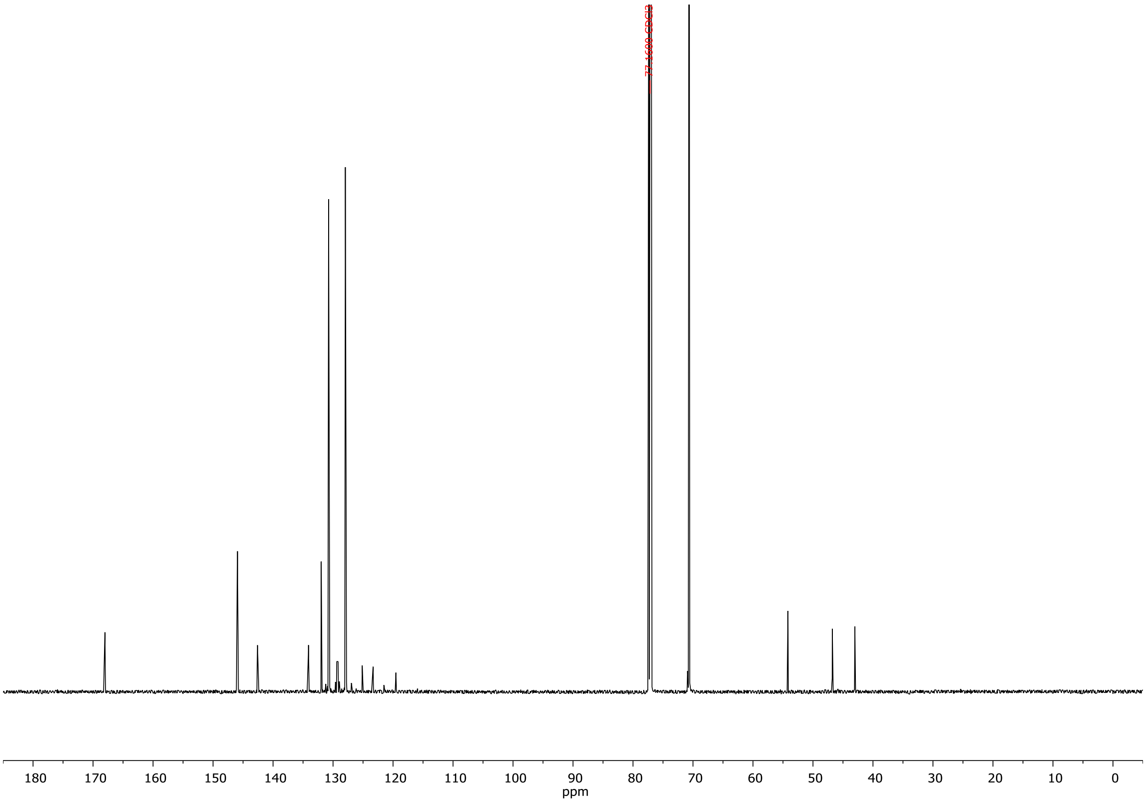

Supplementary Figure 2: ^{13}C NMR ($CDCl_3$, 151 MHz, 295 K) of **6**.

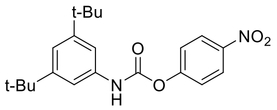

7

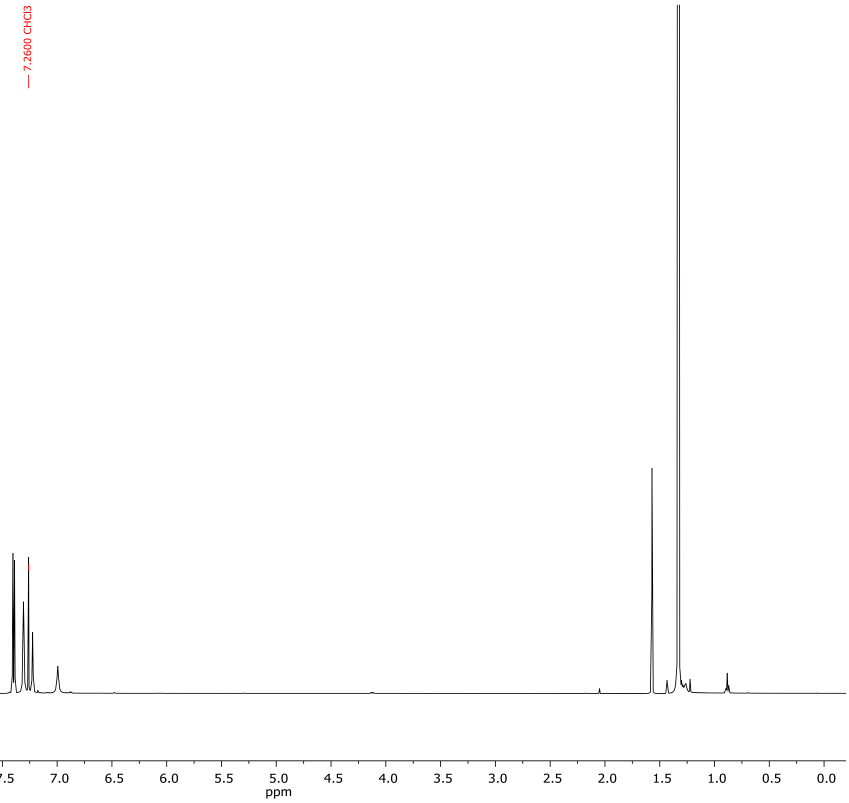

Supplementary Figure 3: ^{1}H NMR ($CDCl_3$, 600 MHz, 295 K) of **7**.

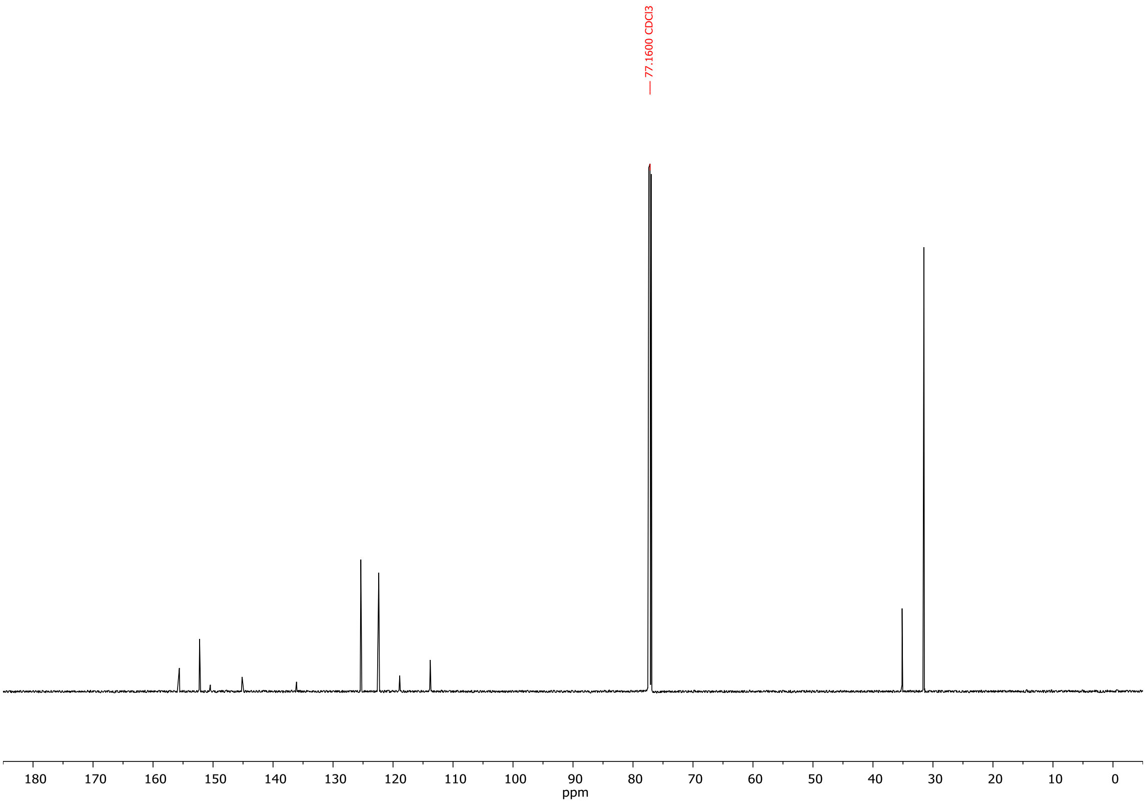

Supplementary Figure 4: ^{13}C NMR ($CDCl_3$, 151 MHz, 295 K) of **7**.

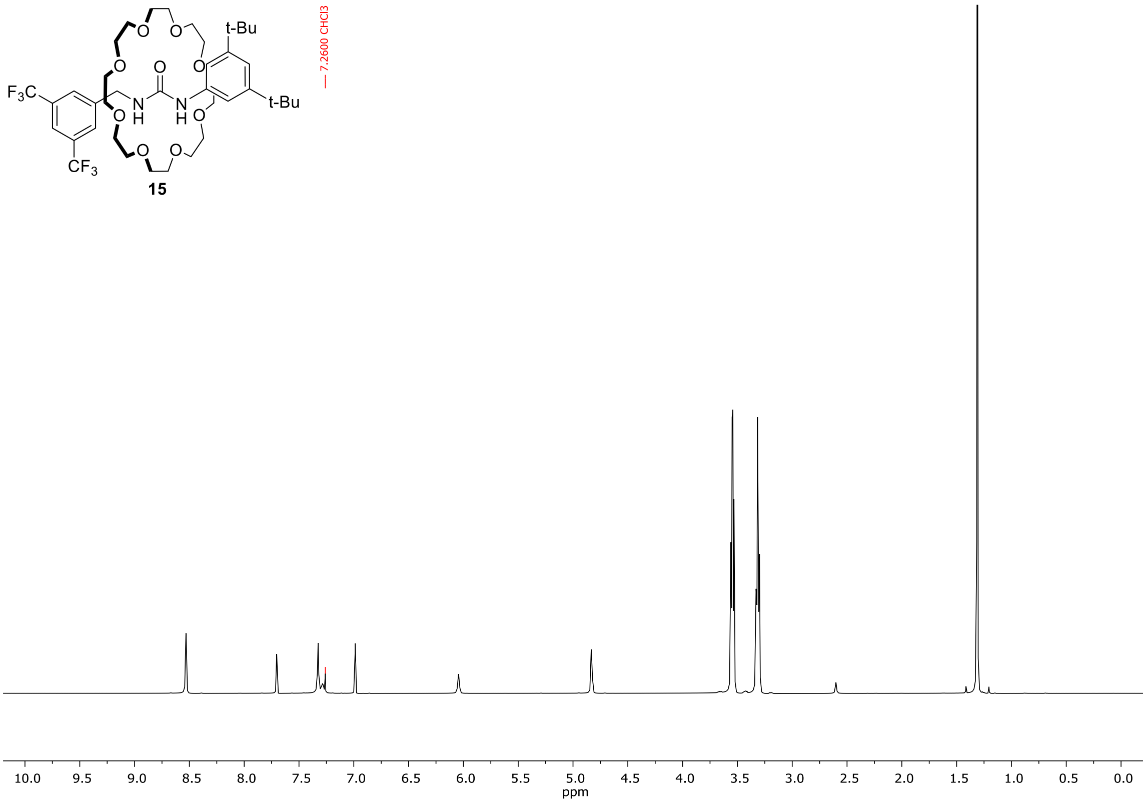

Supplementary Figure 5: ^{1}H NMR ($CDCl_3$, 600 MHz, 295 K) of **15**.

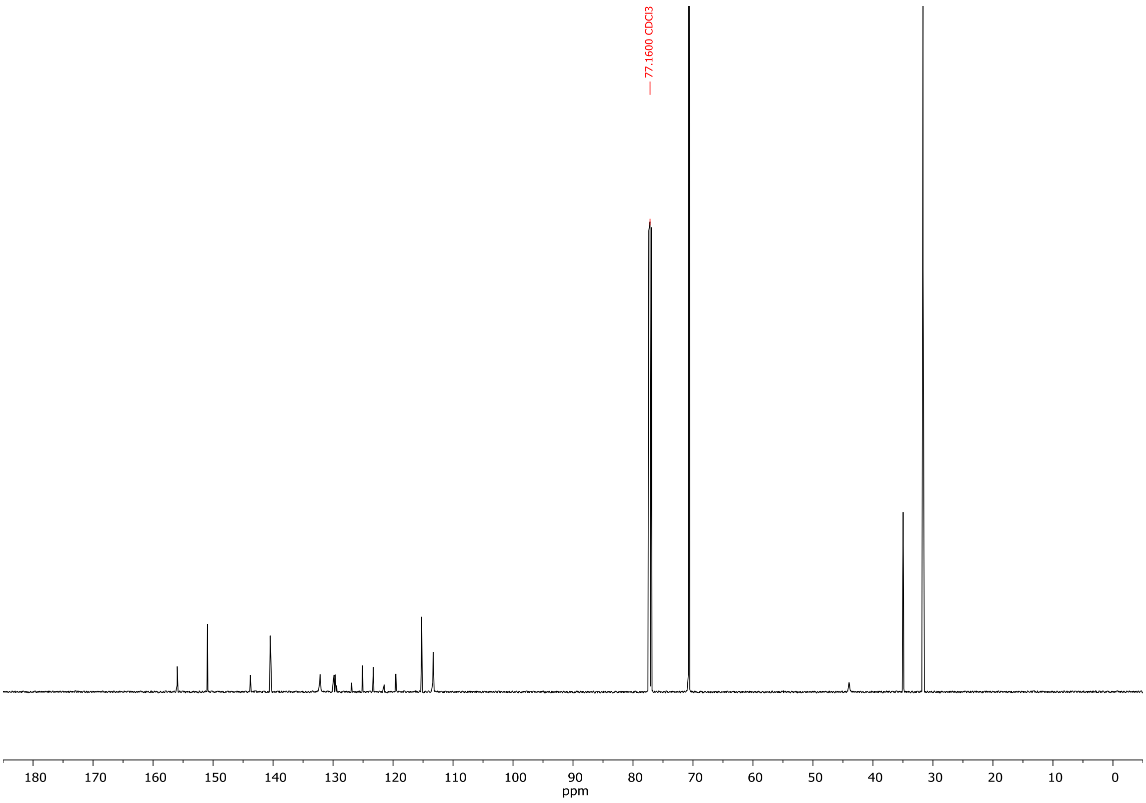

Supplementary Figure 6: ^{13}C NMR ($CDCl_3$, 151 MHz, 295 K) of **15**.

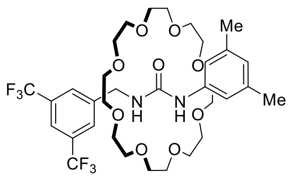

16

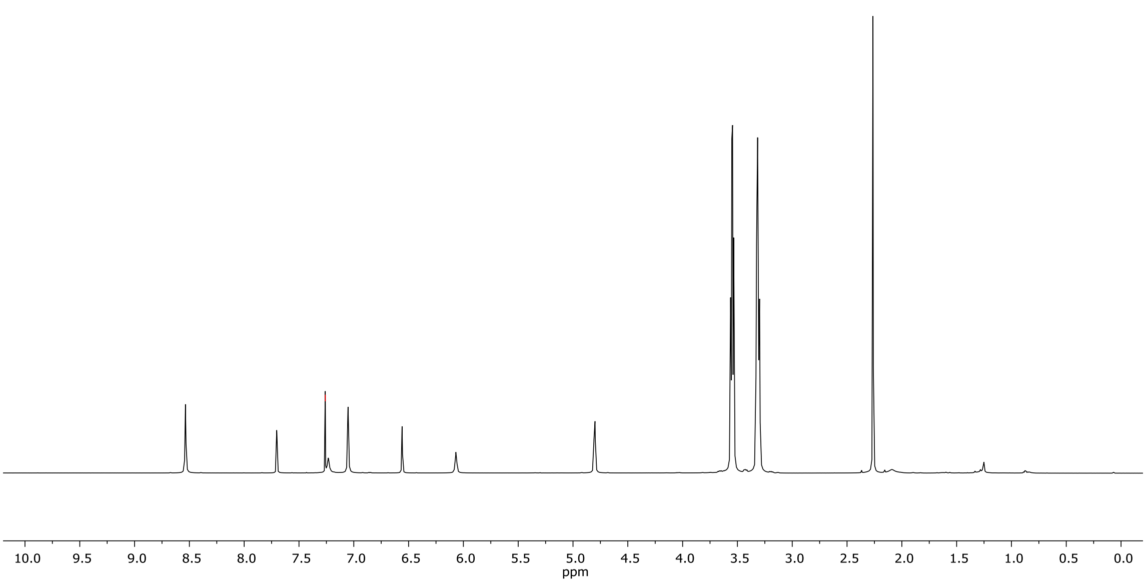

Supplementary Figure 7: ^{1}H NMR ($CDCl_3$, 600 MHz, 295 K) of **16**.

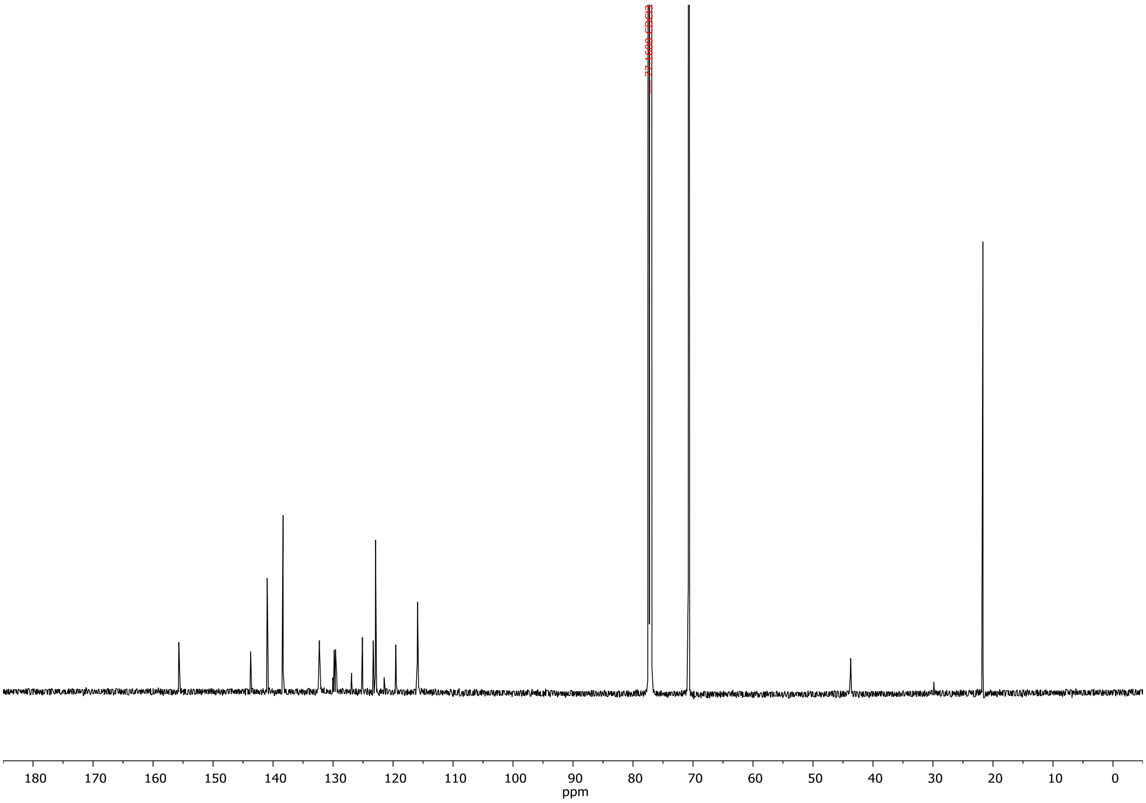

Supplementary Figure 8: ^{13}C NMR ($CDCl_3$, 151 MHz, 295 K) of **16**.

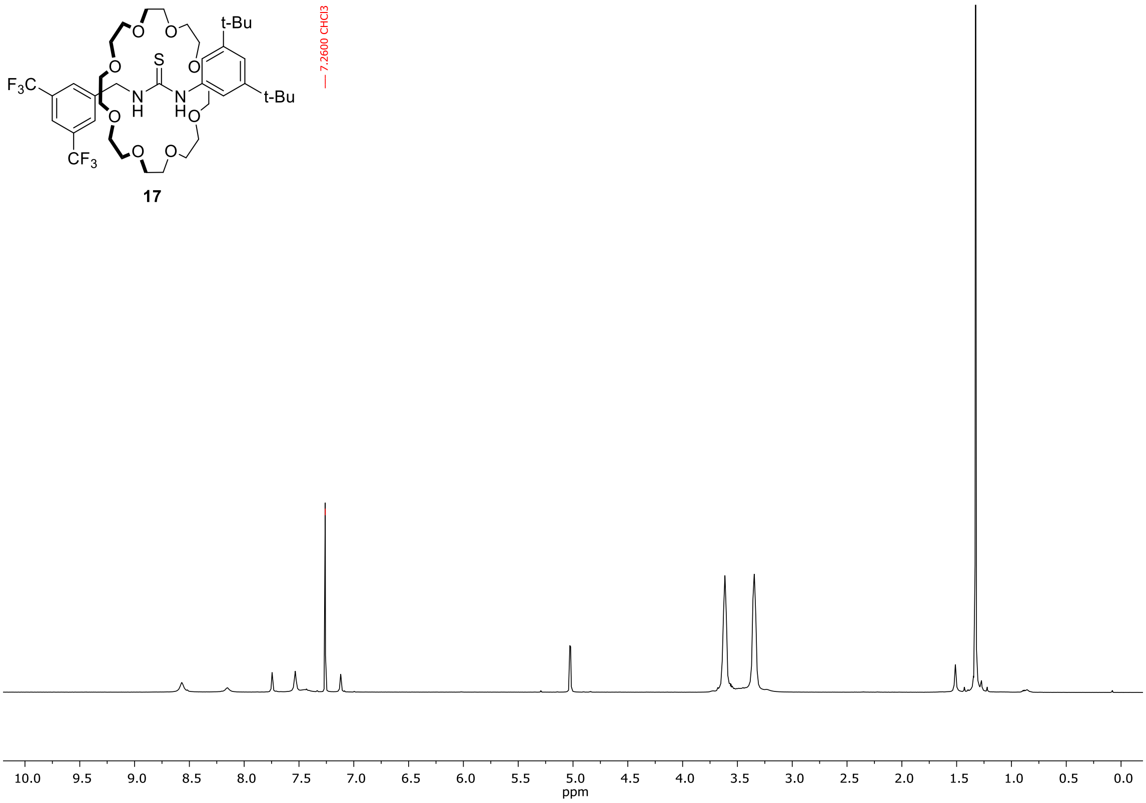

Supplementary Figure 9: ^{1}H NMR ($CDCl_3$, 600 MHz, 325 K) of **17**.

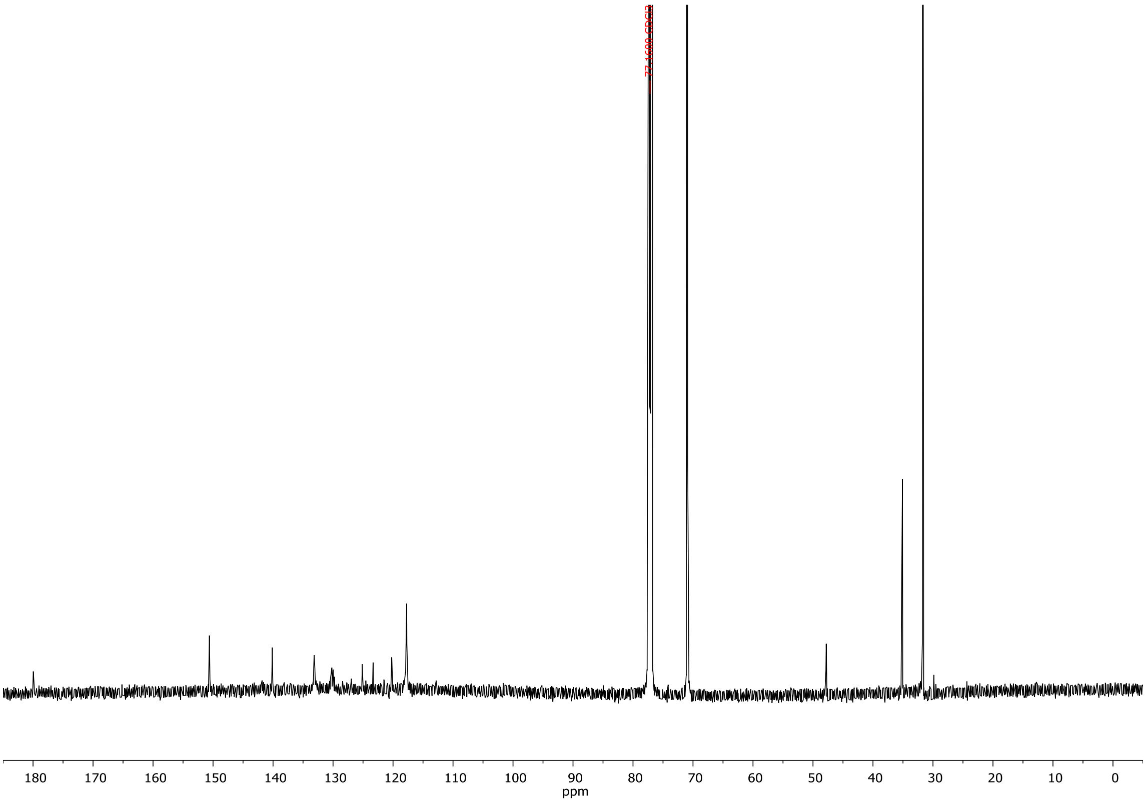

Supplementary Figure 10: ^{13}C NMR ($CDCl_3$, 151 MHz, 325 K) of **17**.

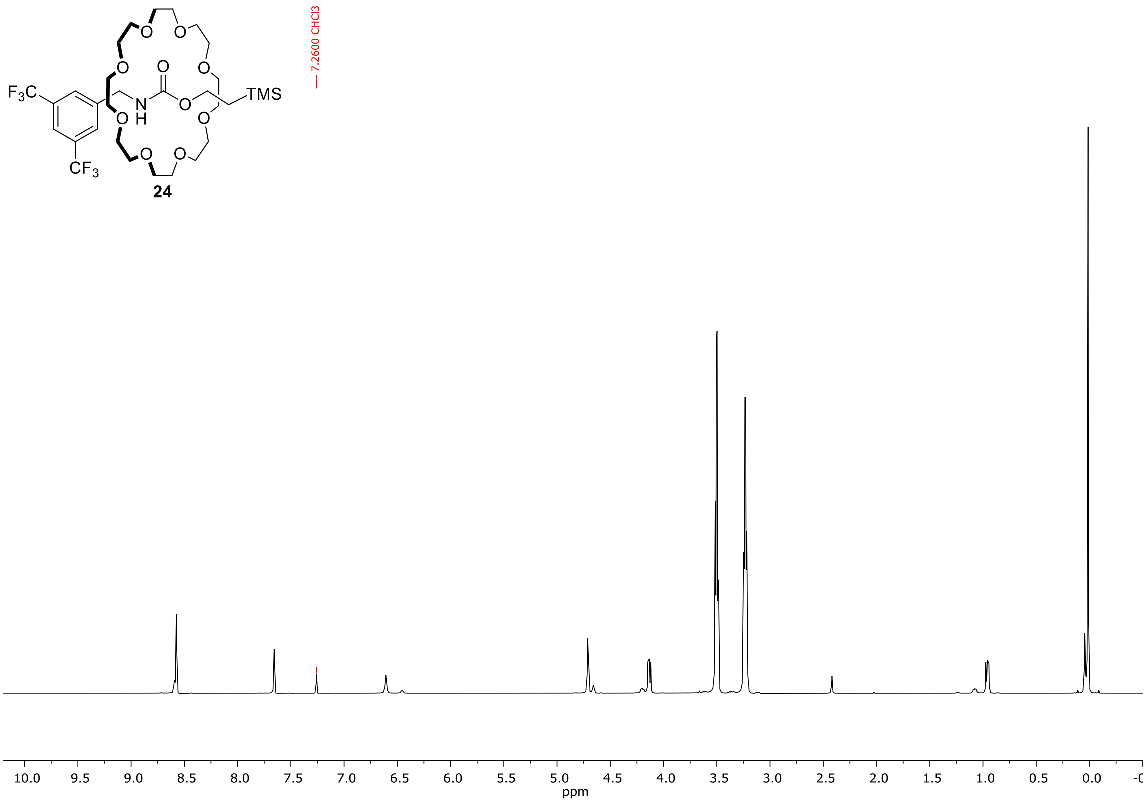

Supplementary Figure 11: ^{1}H NMR ($CDCl_3$, 600 MHz, 295 K) of **18**.

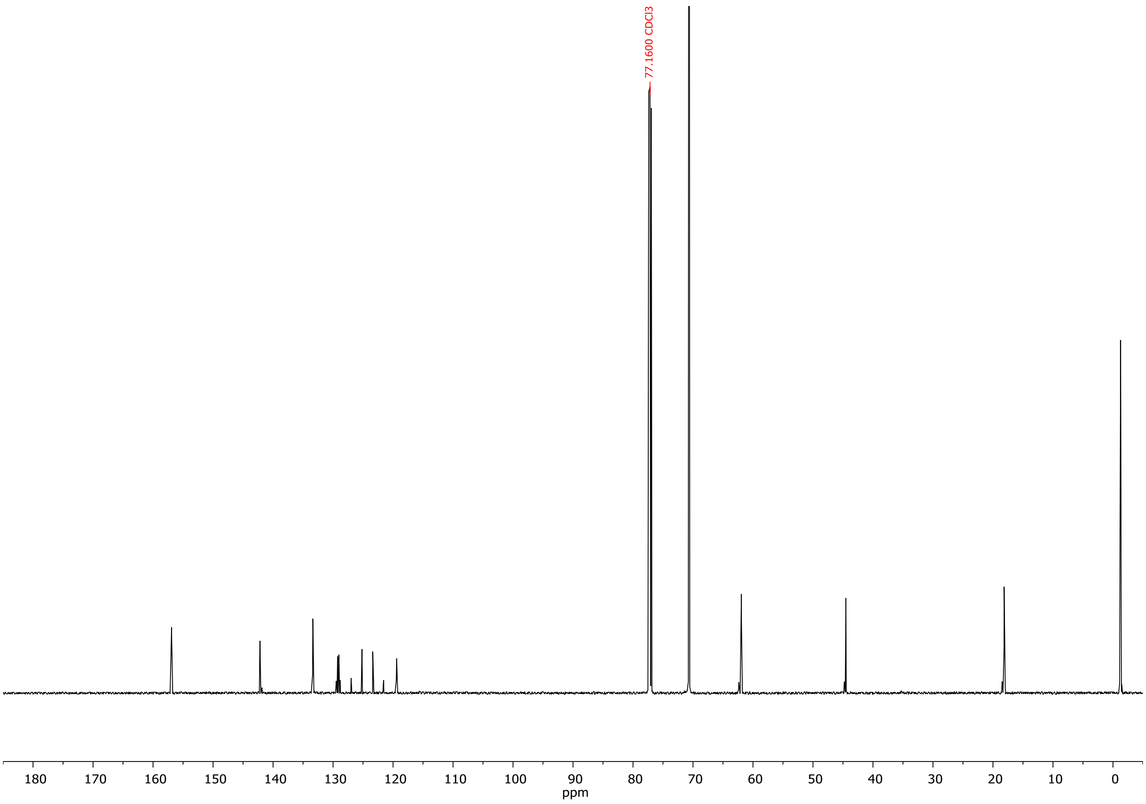

Supplementary Figure 12: ^{13}C NMR ($CDCl_3$, 151 MHz, 295 K) of **18**.

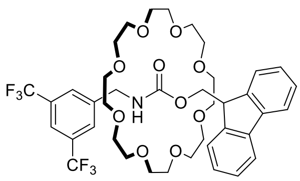

19

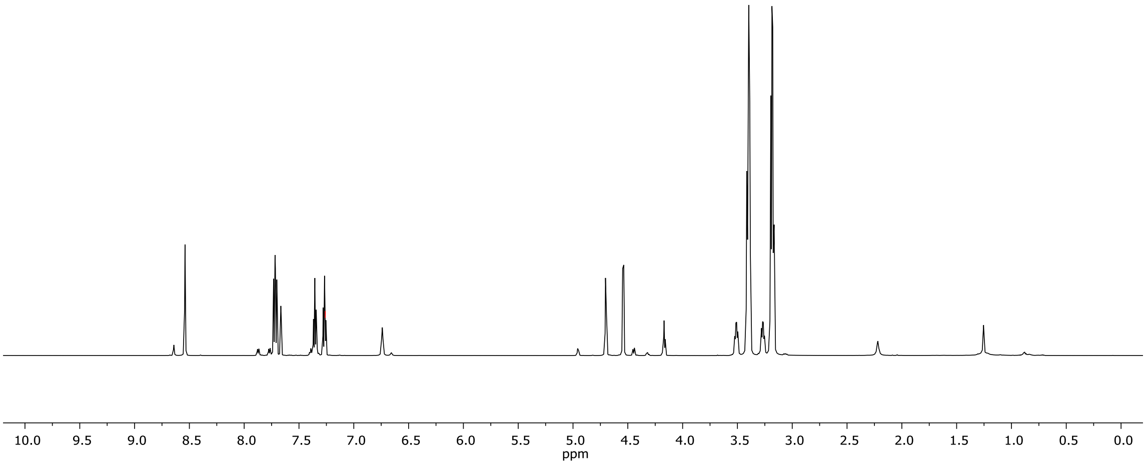

Supplementary Figure 13: ^{1}H NMR ($CDCl_3$, 600 MHz, 295 K) of **19**.

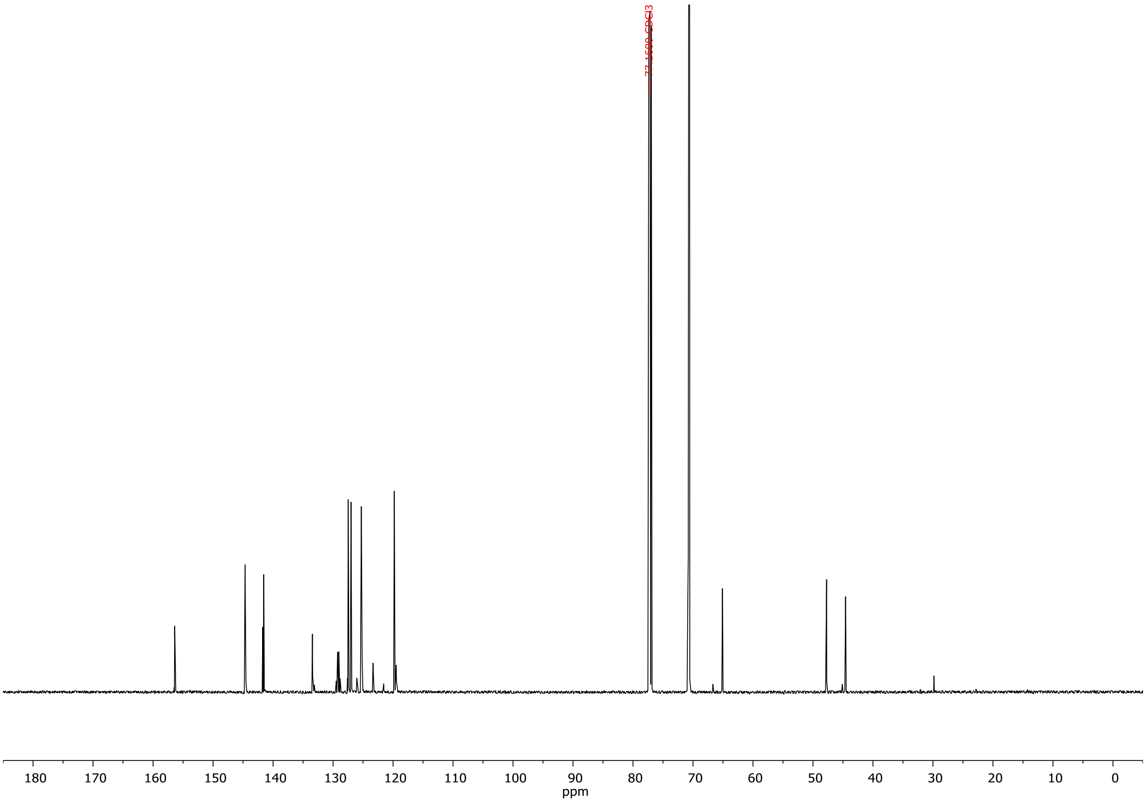

Supplementary Figure 14: ^{13}C NMR ($CDCl_3$, 151 MHz, 295 K) of **19**.

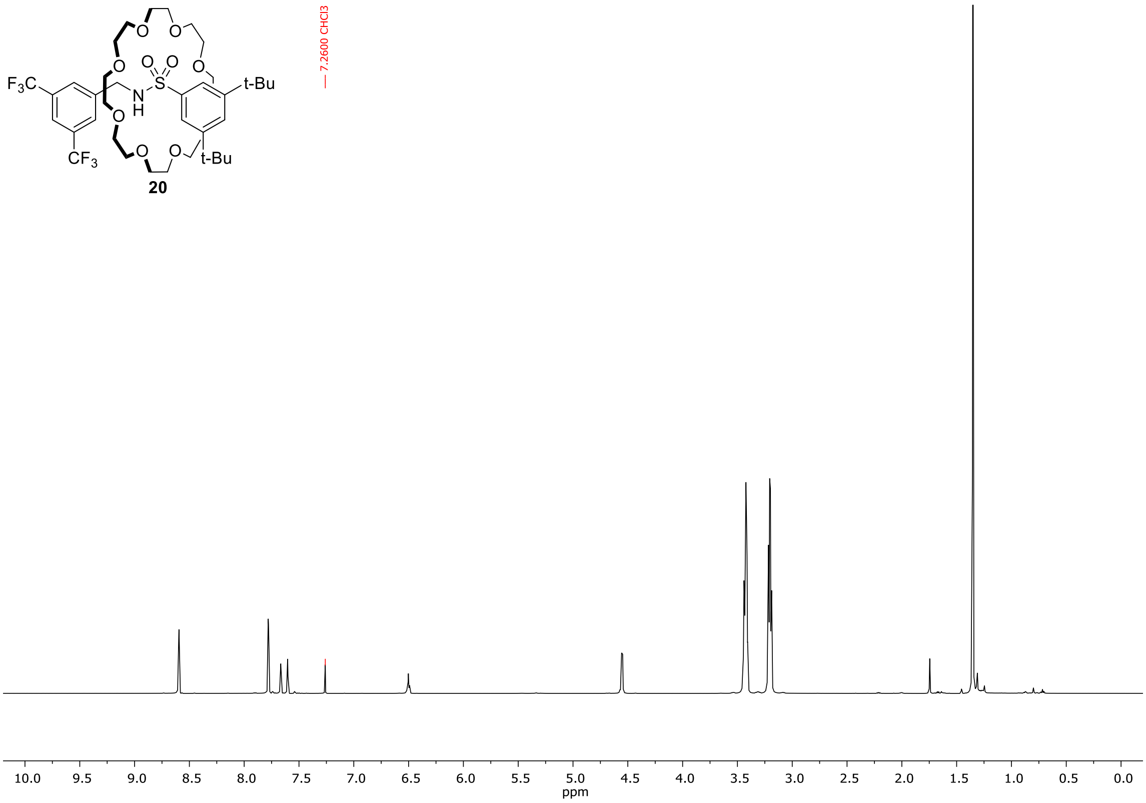

Supplementary Figure 15: ^{1}H NMR ($CDCl_3$, 600 MHz, 295 K) of **20**.

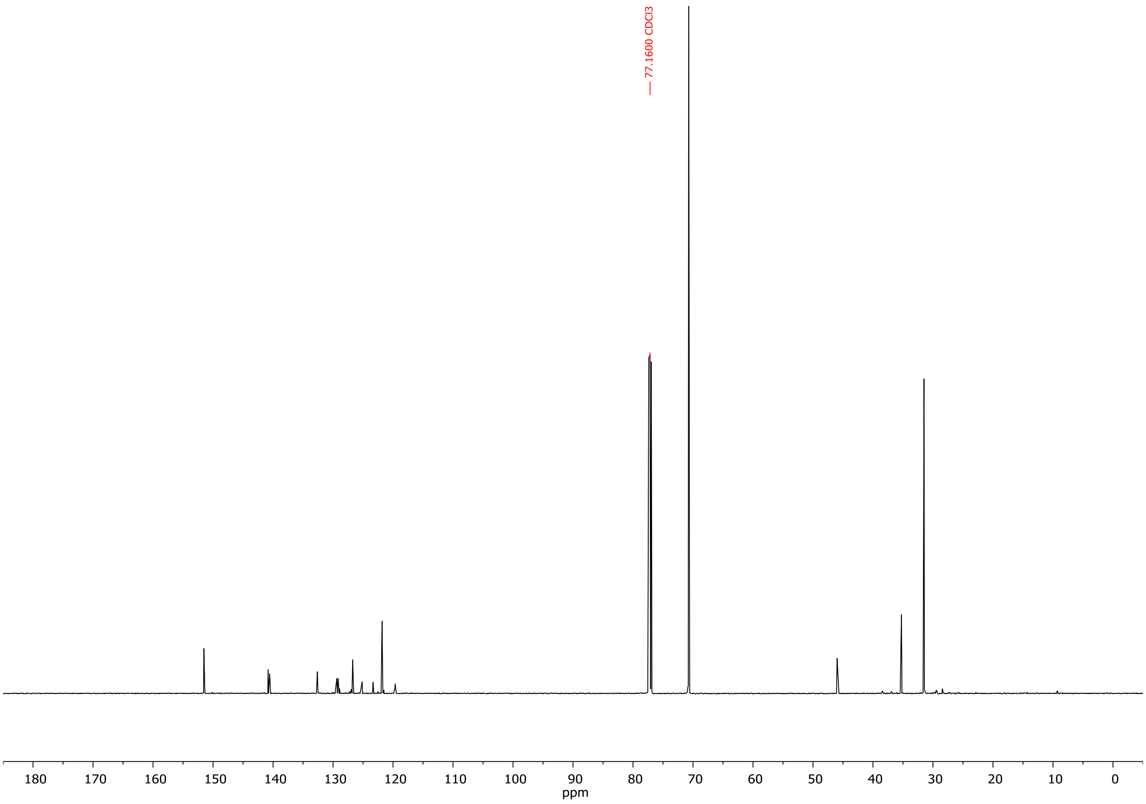

Supplementary Figure 16: ^{13}C NMR ($CDCl_3$, 151 MHz, 295 K) of **20**.

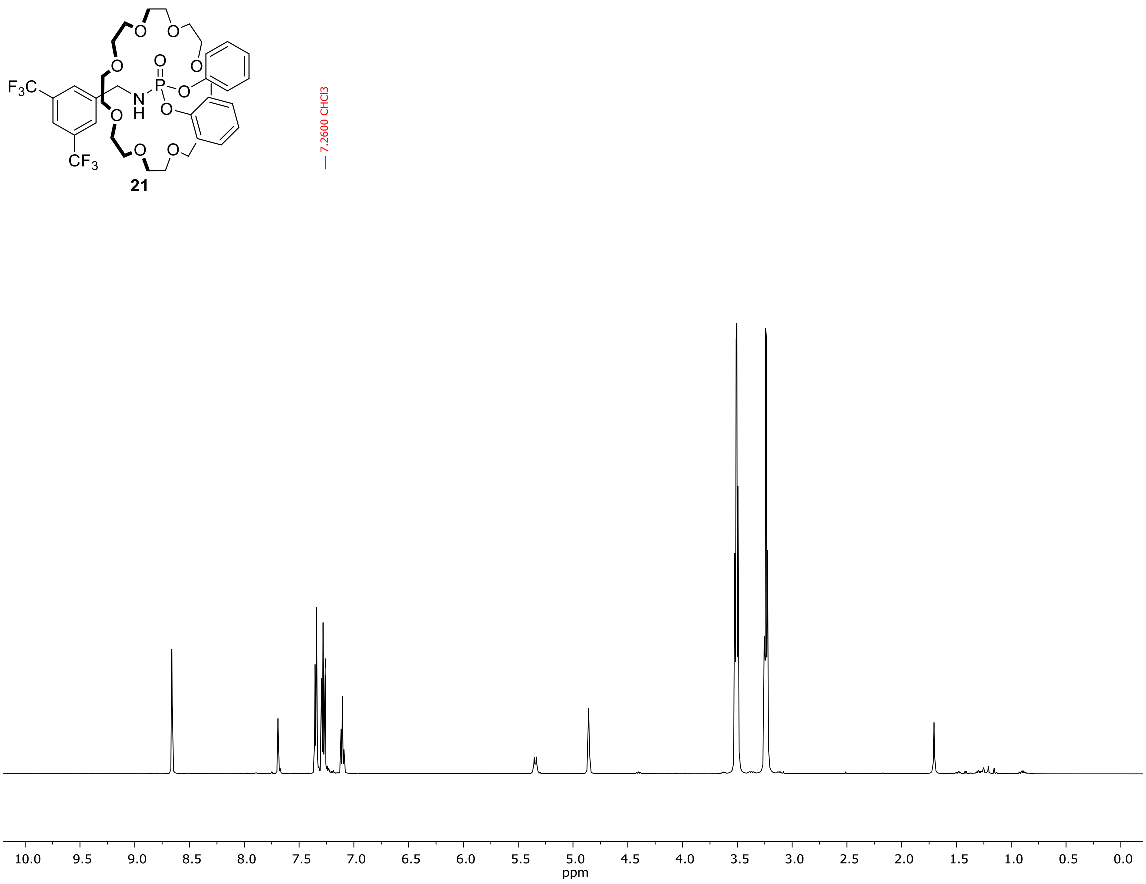

Supplementary Figure 17: ^{1}H NMR ($CDCl_3$, 600 MHz, 295 K) of **21**.

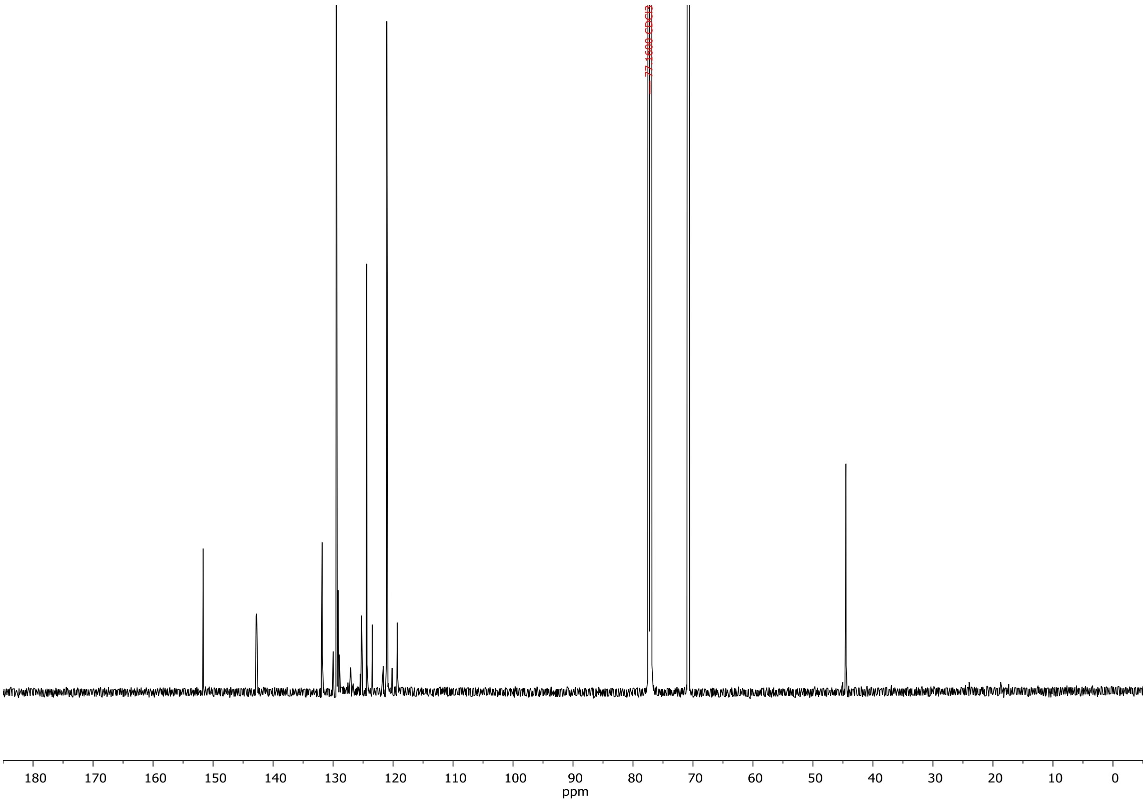

Supplementary Figure 18: ^{13}C NMR ($CDCl_3$, 151 MHz, 295 K) of **21**.

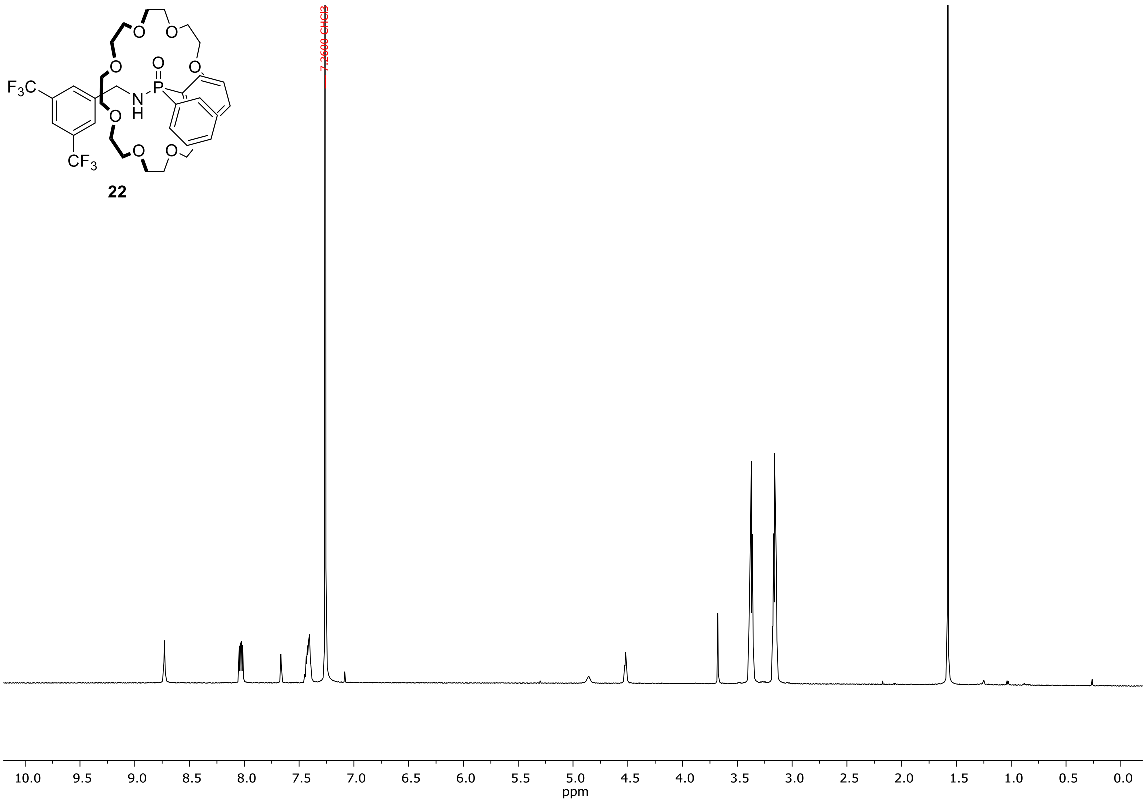

Supplementary Figure 19: ^{1}H NMR ($CDCl_3$, 600 MHz, 295 K) of **22**. Contains a trace of 24-crown-8 at 3.68 ppm.

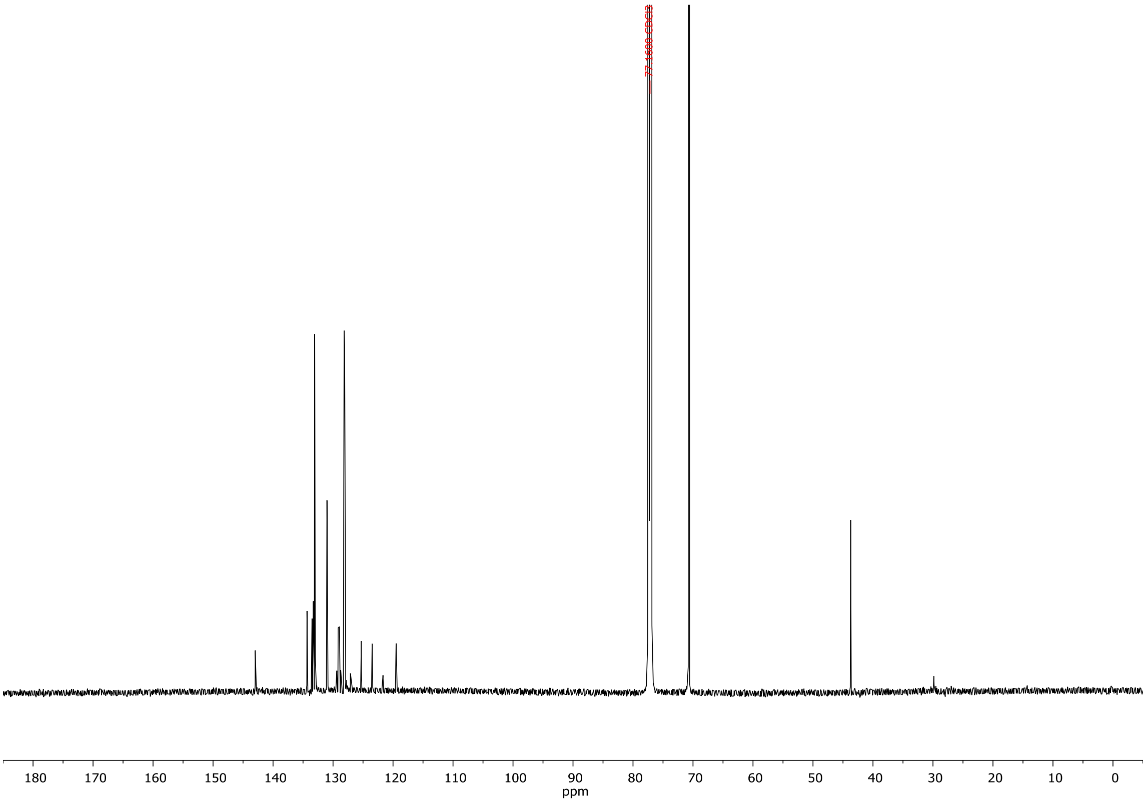

Supplementary Figure 20: ^{13}C NMR ($CDCl_3$, 151 MHz, 295 K) of **22**.

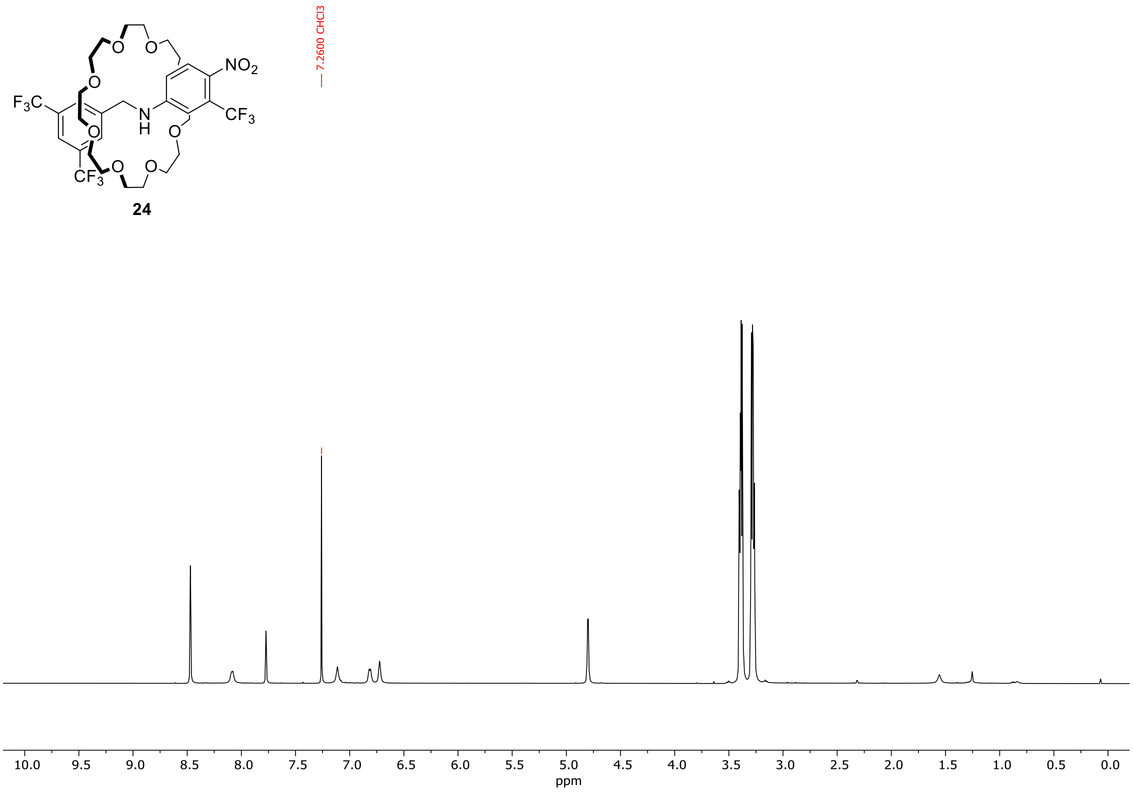

Supplementary Figure 21: ^{1}H NMR ($CDCl_3$, 600 MHz, 295 K) of **24**.

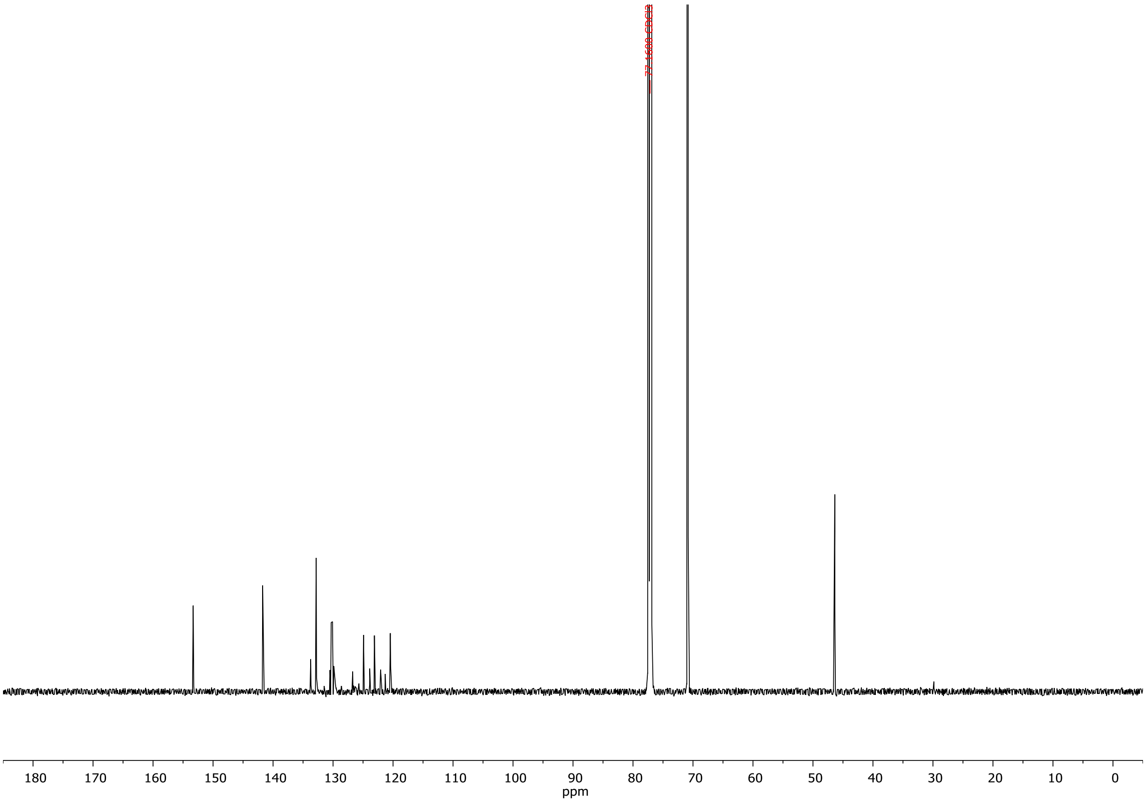

Supplementary Figure 22: ^{13}C NMR ($CDCl_3$, 151 MHz, 295 K) of **24**.

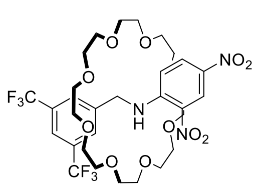

26

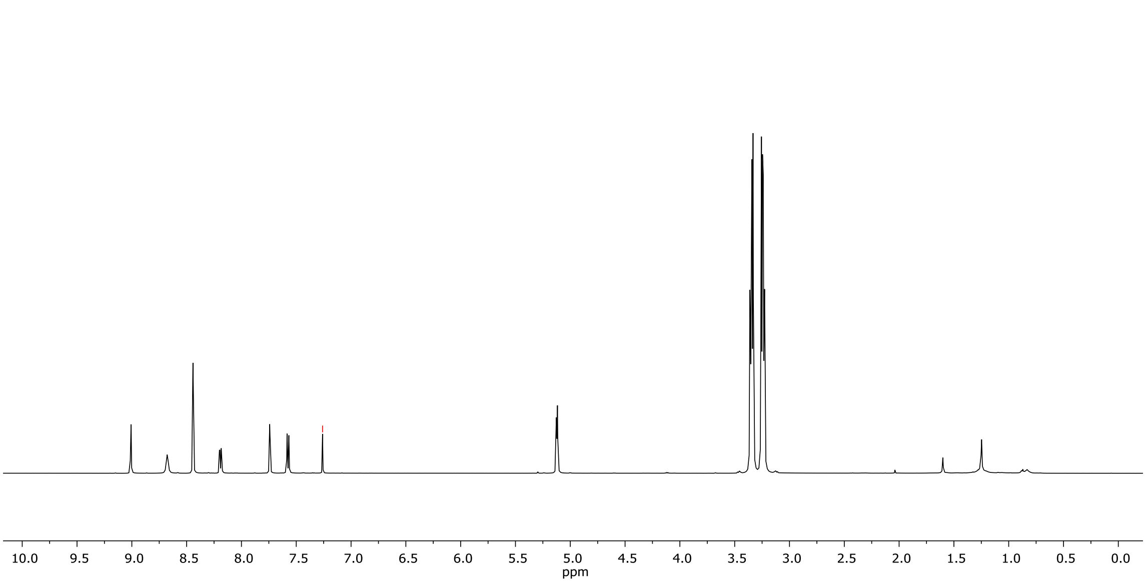

Supplementary Figure 23: ^{1}H NMR ($CDCl_3$, 600 MHz, 295 K) of **26**.

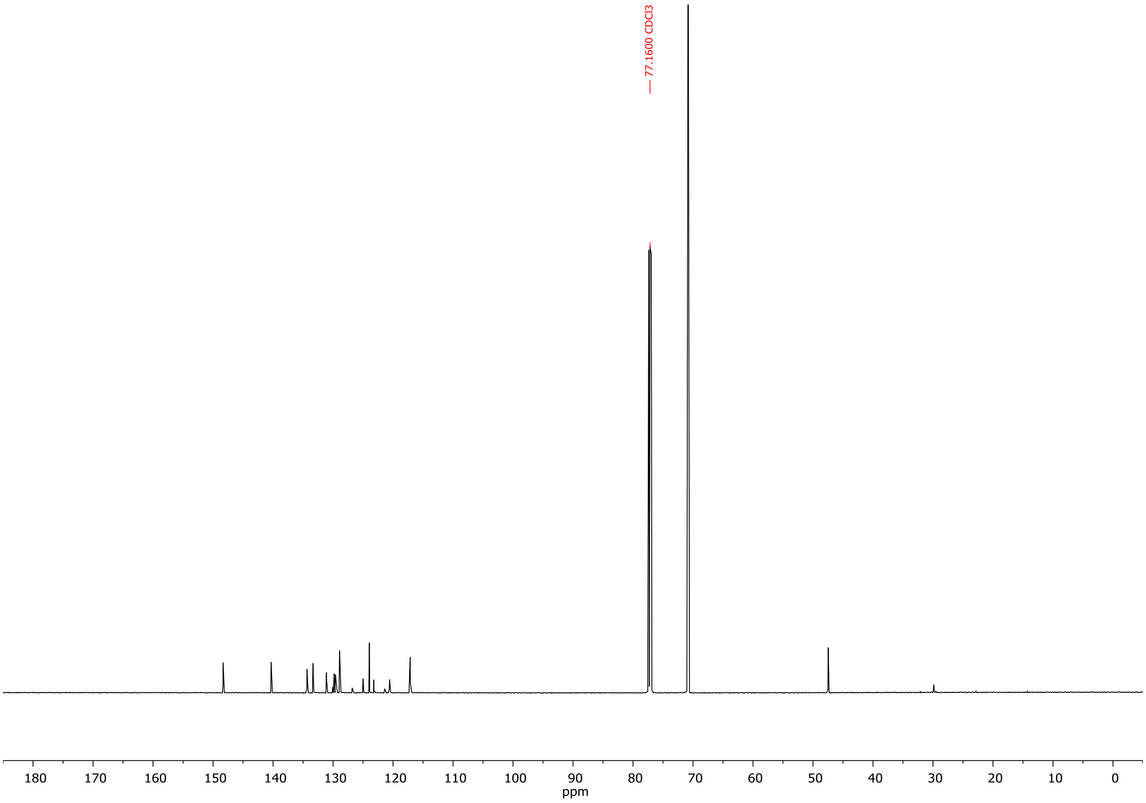

Supplementary Figure 24: ^{13}C NMR ($CDCl_3$, 151 MHz, 295 K) of **26**.

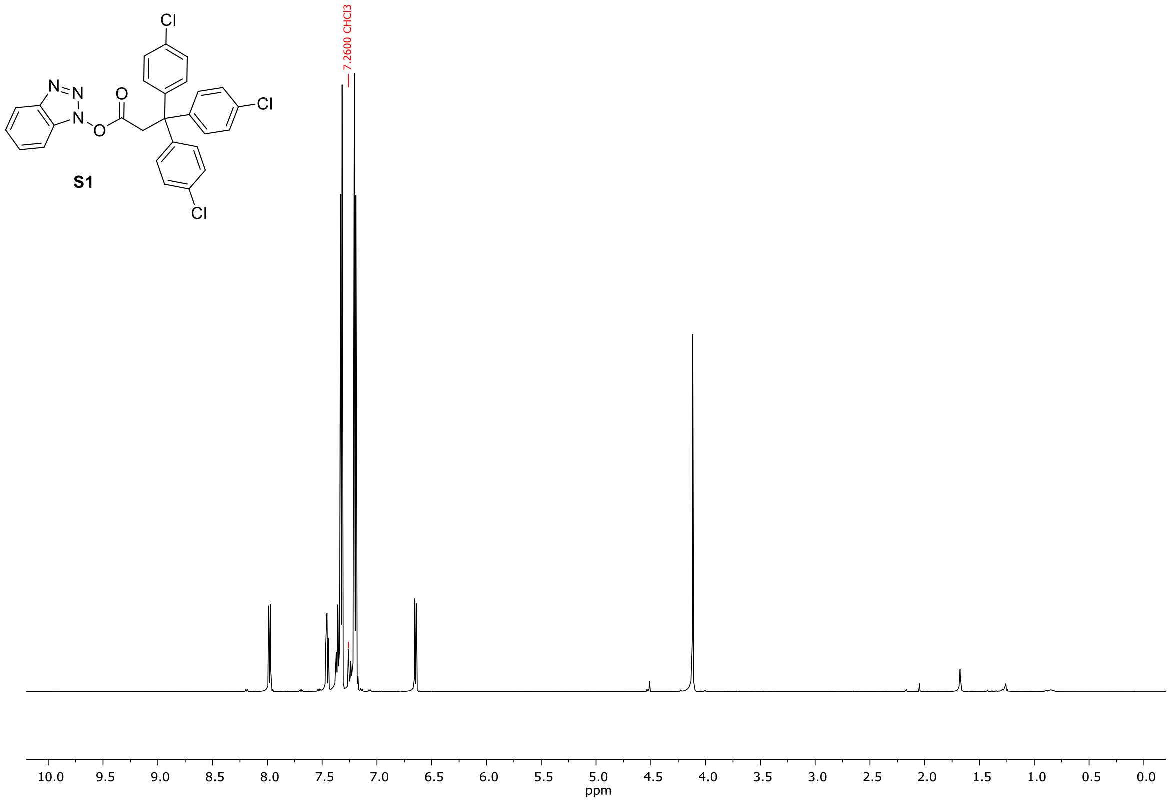

Supplementary Figure 25: ^{1}H NMR ($CDCl_3$, 600 MHz, 295 K) of **S1**.

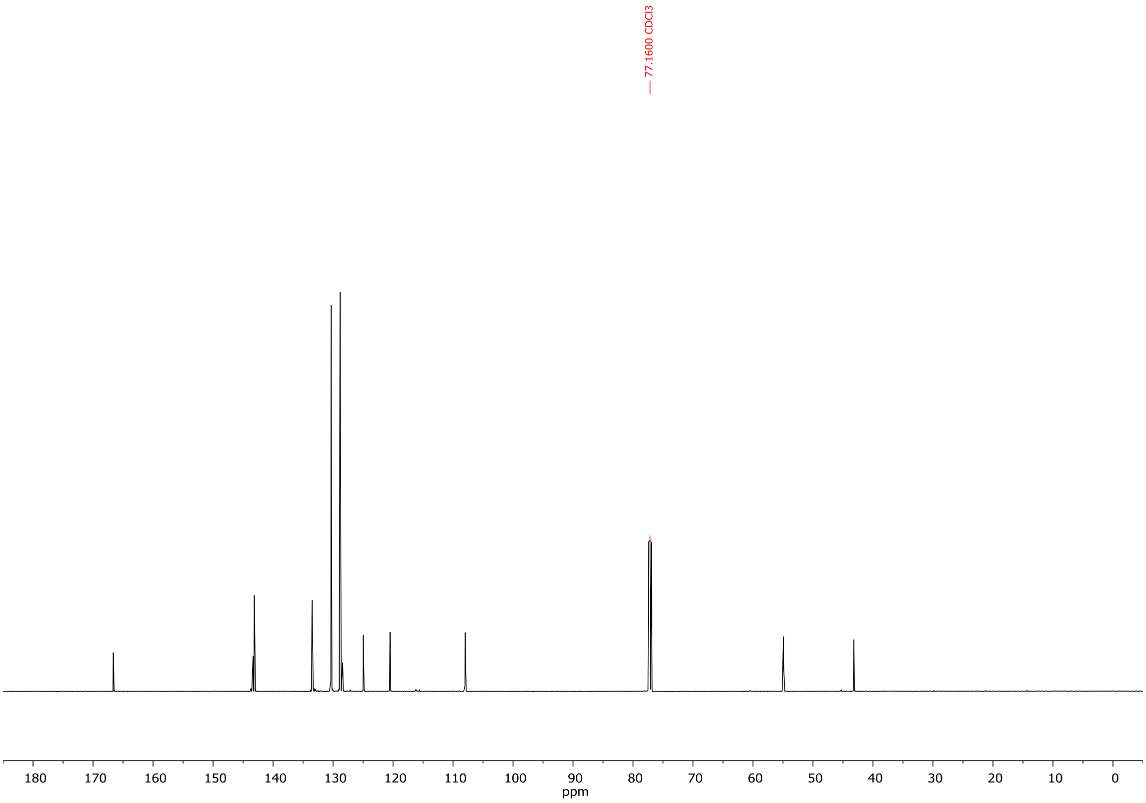

Supplementary Figure 26: ^{13}C NMR ($CDCl_3$, 151 MHz, 295 K) of **S1**.

6 Single Crystal X-Ray Diffraction

Data collection: X-ray diffraction data for compound **4**, **6**, **15, 17, 19-22** were collected on a dual source Rigaku FR-X rotating anode diffractometer using CuK_α and MoK_α wavelength, at a temperature of 150 K and 100 K, respectively.

Data processing: Data were processed and reduced using CrysAlisPro 171.40.26a. Absorption correction was performed using empirical methods (SCALE3 ABSPACK) based upon symmetry-equivalent reflections combined with measurements at different azimuthal angles.[5] The structure was solved using ShelXT 2015 and refined against all F^2 values using Shelxl 2018/1 implemented through Olex2 v1.2.10.[6,7]

Refinement details: The thread and ring in compound **21** were both found to be highly disordered. The disordered parts were defined as residues and the atoms for each residue were restrained to have similar 1,2- and 1,3- atomic distances. The extensive modelling of the disorder of the thread is due to the additional components that are evident from the large electron density regions in the difference map around the phosphorus when this disorder is not taken into account. In order to refine sensible geometries, the third component had to be almost entirely modelled in addition to the first two parts, even though the occupancy is particularly low; the model would have been incorrect if just the phosphorus was modelled to account for the remaining residual peak in the difference map. The additional modelling has resulted in a low data to parameter ratio of < 8. This is regrettable, but modelling part of the structure as isotropic to account for this was deemed inappropriate. In addition, a strong neighbouring similar thermal parameter restraint was applied to the model due to the large degree of disorder within the structure. Without these restraints the thermal parameters do not refine to sensible values.

CCDC 1907190-1907197 contains the supplementary crystallographic data for this paper. These data can be obtained free of charge *via* www.ccdc.cam.ac.uk/conts/retrieving.html (or from the Cambridge Crystallographic Data Centre, 12 Union Road, Cambridge CB21EZ, UK; fax: (+44)1223-336-033; or deposit@ccdc.cam.ac.uk).

Supplementary Table 4. Crystallographic information for compounds **4**, **6**, **15** and **17**

Identification code	**4**	**6**	**15**	**17**
Empirical formula	$C_{52}H_{70}Cl_3NO_9$	$C_{46}H_{52}Cl_3F_6NO_9$	$C_{40}H_{60}F_6N_2O_9$	$C_{40}H_{60}F_6N_2O_8S$
Formula weight	959.44	983.23	826.90	842.96
Temperature/K	293.15	150.01(10)	150.00(10)	99.8(6)
Crystal system	triclinic	monoclinic	monoclinic	triclinic
Space group	P-1	$P2_1/c$	$P2_1/n$	P-1
a/Å	12.3177(4)	11.93510(10)	10.89980(10)	11.2738(12)
b/Å	12.8612(5)	12.67690(10)	14.8877(2)	13.7837(16)
c/Å	16.2830(5)	31.8164(3)	27.0223(3)	14.6089(6)
α/°	81.981(3)	90	90	90.412(7)
β/°	83.696(3)	97.8850(10)	100.8940(10)	92.199(6)
γ/°	89.296(3)	90	90	102.157(10)
Volume/Å^3	2538.88(15)	4768.31(7)	4305.96(9)	2217.3(4)
Z	2	4	4	2
ρ_{calc}g/cm^3	1.255	1.370	1.276	1.263
μ/mm^{-1}	2.076	2.415	0.902	1.296
F(000)	1024.0	2048.0	1760.0	896.0
Crystal size/mm^3	0.18 × 0.16 × 0.03	0.4 × 0.38 × 0.2	0.56 × 0.5 × 0.45	0.226 × 0.118 × 0.029
Radiation	CuKα (λ = 1.54184)	CuKα (λ = 1.54184)	CuKα (λ = 1.54184)	CuKα (λ = 1.54184)
2Θ range for data collection/°	5.514 to 133.192	5.608 to 133.178	6.662 to 141.878	6.056 to 138.982
Index ranges	-14 ≤ h ≤ 14, -15 ≤ k ≤ 15, -15 ≤ l ≤ 17	-12 ≤ h ≤ 14, -15 ≤ k ≤ 15, -37 ≤ l ≤ 37	-13 ≤ h ≤ 13, -17 ≤ k ≤ 13, -32 ≤ l ≤ 32	-13 ≤ h ≤ 13, -16 ≤ k ≤ 16, -17 ≤ l ≤ 17
Reflections collected	48848	43983	16799	13589
Independent reflections	8580 [R_{int} = 0.0361, R_{sigma} = 0.0242]	7784 [R_{int} = 0.0313, R_{sigma} = 0.0175]	7993 [R_{int} = 0.0230, R_{sigma} = 0.0231]	13589 [R_{int} = 0.0700, R_{sigma} = 0.0540]
Data/restraints/parameters	8580/0/592	7784/226/661	7993/145/576	13589/39/552
Goodness-of-fit on F^2	1.056	1.039	1.001	1.046
Final R indexes [I>=2σ (I)]	R_1 = 0.0386, wR_2 = 0.0986	R_1 = 0.0518, wR_2 = 0.1282	R_1 = 0.0619, wR_2 = 0.1593	R_1 = 0.0798, wR_2 = 0.2272
Final R indexes [all data]	R_1 = 0.0430, wR_2 = 0.1014	R_1 = 0.0525, wR_2 = 0.1287	R_1 = 0.0658, wR_2 = 0.1623	R_1 = 0.0953, wR_2 = 0.2361
Largest diff. peak/hole / e Å^{-3}	0.38/-0.46	0.99/-0.51	0.73/-0.71	0.67/-0.39

Supplementary Table 5. Crystallographic information for compounds **19**-**22**

Identification code	**19**	**20**	**21**	**22**
Empirical formula	$C_{40}H_{49}F_6NO_{10}$	$C_{41}H_{64}F_6NO_{10.5}S$	$C_{37}H_{48}F_6NO_{11}P$	$C_{37}H_{48}F_6NO_9P$
Formula weight	817.80	884.99	827.73	795.73
Temperature/K	150.00(10)	224.97(10)	150.00(10)	150.00(10)
Crystal system	monoclinic	triclinic	monoclinic	orthorhombic
Space group	$P2_1/c$	P-1	$P2_1/c$	$Pca2_1$
a/Å	8.85690(10)	10.6949(3)	9.14895(15)	17.1007(4)
b/Å	12.56130(10)	13.8111(5)	37.4344(7)	11.6027(3)
c/Å	36.6448(2)	17.1512(6)	12.4568(3)	38.7645(10)
α/°	90	73.120(3)	90	90
β/°	91.6800(10)	84.494(3)	110.283(2)	90
γ/°	90	86.392(3)	90	90
Volume/Å^3	4075.14(6)	2411.40(14)	4001.72(14)	7691.4(3)
Z	4	2	4	8
ρ_{calc}g/cm^3	1.333	1.219	1.374	1.374
μ/mm^{-1}	0.965	0.142	1.374	0.155
F(000)	1720.0	942.0	1736.0	3344.0
Crystal size/mm^3	0.58 × 0.3 × 0.25	0.35 × 0.32 × 0.3	0.348 × 0.200 × 0.081	0.6 × 0.35 × 0.2
Radiation	CuKα (λ = 1.54184)	MoKα (λ = 0.71073)	CuKα (λ = 1.54184)	MoKα (λ = 0.71073)
2Θ range for data collection/°	4.824 to 153.998	3.084 to 61.356	4.722 to 153.672	3.51 to 61.56
Index ranges	-10 ≤ h ≤ 11, -15 ≤ k ≤ 11, -45 ≤ l ≤ 43	-15 ≤ h ≤ 14, -19 ≤ k ≤ 18, -21 ≤ l ≤ 23	-10 ≤ h ≤ 11, -46 ≤ k ≤ 43, -12 ≤ l ≤ 15	-18 ≤ h ≤ 23, -14 ≤ k ≤ 16, -47 ≤ l ≤ 54
Reflections collected	43050	31811	22610	72185
Independent reflections	8499 [R_{int} = 0.0233, R_{sigma} = 0.0118]	11965 [R_{int} = 0.0512, R_{sigma} = 0.0758]	8148 [R_{int} = 0.0270, R_{sigma} = 0.0349]	18789 [R_{int} = 0.1169, R_{sigma} = 0.1139]
Data/restraints/parameters	8499/27/514	11965/280/635	8148/3725/1102	18789/1/974
Goodness-of-fit on F^2	1.069	1.037	1.081	1.085
Final R indexes [I>=2σ (I)]	R_1 = 0.0628, wR_2 = 0.1572	R_1 = 0.0921, wR_2 = 0.2391	R_1 = 0.0660, wR_2 = 0.2270	R_1 = 0.0596, wR_2 = 0.1470
Final R indexes [all data]	R_1 = 0.0644, wR_2 = 0.1588	R_1 = 0.1405, wR_2 = 0.2688	R_1 = 0.0810, wR_2 = 0.2541	R_1 = 0.0872, wR_2 = 0.1649
Largest diff. peak/hole / e Å^{-3}	0.60/-0.60	0.58/-0.34	0.38/-0.49	0.75/-0.72

7 Supplementary References

1 Fielden, S. D. P., Leigh, D. A., McTernan, C. T., Pérez-Saavedra, B. & Vitorica-Yrezabal, I. J. Spontaneous assembly of rotaxanes from a primary amine, crown ether and electrophile. *J. Am. Chem. Soc*. **140**, 6049–6052 (2018).

2 Hannam, J. S., Kidd, T. J., Leigh, D. A. & Wilson, A. J. “Magic Rod” Rotaxanes: the hydrogen bond-directed synthesis of molecular shuttles under thermodynamic control. *Org. Lett.* **5**, 1907–1910 (2003).

3 Fu, Z., Yuan, W., Chen, N., Yang, Z. & Xu, J. $Na_2S_2O_8$-mediated efficient synthesis of isothiocyanates from primary amines in water. *Green Chem.* **20**, 4484–4891 (2018).

4 Guthrie, R. D., Thang, S. Selective sulfonylating agents. *Aust. J. Chem.* **40**, 2133–2136 (1987).

5 *Rigaku Oxford Diffraction*, CrysAlisPro Software system, version 1.171.40.26a, Rigaku Corporation, Oxford, UK, (2017).

6 a) Sheldrick, G. M. Integrated space-group and crystal-structure determination. *Acta Cryst.* **A71**, 3–8 (2015); b) Sheldrick, G. M. Crystal structure refinement with SHELXL. *Acta Cryst*. **C17**, 3–8 (2015).

7 Dolomanov, O. V., Bourhis, L. J., Gildea, R. J., Howard, J. A. K. & Puschmann, H. OLEX2: a complete structure solution, refinement and analysis program. *J. Appl. Cryst*. **42**, 339–341 (2009).
